# Modulation of Human Macrophage Responses to *Mycobacterium tuberculosis* by Silver Nanoparticles of Different Size and Surface Modification

**DOI:** 10.1371/journal.pone.0143077

**Published:** 2015-11-18

**Authors:** Srijata Sarkar, Bey Fen Leo, Claudia Carranza, Shu Chen, Cesar Rivas-Santiago, Alexandra E. Porter, Mary P. Ryan, Andrew Gow, Kian Fan Chung, Teresa D. Tetley, Junfeng (Jim) Zhang, Panos G. Georgopoulos, Pamela A. Ohman-Strickland, Stephan Schwander

**Affiliations:** 1 Department of Environmental and Occupational Health School of Public Health, Rutgers University, Piscataway, New Jersey, United States of America; 2 Department of Materials and London Center for Nanotechnology, Imperial College, London, United Kingdom; 3 Department of Mechanical Engineering, University of Malaya, Kuala Lumpur, Malaysia; 4 Department of Microbiology, Instituto Nacional de Enfermedades Respiratorias, Mexico City, Mexico; 5 Department of Toxicology, Ernest Mario School of Pharmacy, Rutgers University, Piscataway, New Jersey, United States of America; 6 National Heart and Lung Institute, Imperial College, London, United Kingdom; 7 Nicholas School of the Environment and Duke Global Health Institute, Duke University, Durham, North Carolina, United States of America; 8 Computational Chemodynamics Laboratory, Environmental and Occupational Health Sciences Institute, Robert Wood Johnson Medical School, Rutgers University, Piscataway, New Jersey, United States of America; 9 Department of Biostatistics, Rutgers University School of Public Health, Piscataway, New Jersey, United States of America; 10 Center for Global Public Health, School of Public Health, Rutgers University, Piscataway, New Jersey, United States of America; University of the Pacific, UNITED STATES

## Abstract

Exposure to silver nanoparticles (AgNP) used in consumer products carries potential health risks including increased susceptibility to infectious pathogens. Systematic assessments of antimicrobial macrophage immune responses in the context of AgNP exposure are important because uptake of AgNP by macrophages may lead to alterations of innate immune cell functions. In this study we examined the effects of exposure to AgNP with different particle sizes (20 and 110 nm diameters) and surface chemistry (citrate or polyvinlypyrrolidone capping) on cellular toxicity and innate immune responses against *Mycobacterium tuberculosis* (*M*.*tb*) by human monocyte-derived macrophages (MDM). Exposures of MDM to AgNP significantly reduced cellular viability, increased *IL8* and decreased *IL10* mRNA expression. Exposure of *M*.*tb*-infected MDM to AgNP suppressed *M*.*tb*-induced expression of *IL1B*, *IL10*, and *TNFA* mRNA. Furthermore, *M*.*tb*-induced IL-1β, a cytokine critical for host resistance to *M*.*tb*, was inhibited by AgNP but not by carbon black particles indicating that the observed immunosuppressive effects of AgNP are particle specific. Suppressive effects of AgNP on the *M*.*tb*-induced host immune responses were in part due to AgNP-mediated interferences with the TLR signaling pathways that culminate in the activation of the transcription factor NF-κB. AgNP exposure suppressed *M*.*tb*-induced expression of a subset of NF-κB mediated genes (*CSF2*, *CSF3*, *IFNG*, *IL1A*, *IL1B*, *IL6*, *IL10*, *TNFA*, *NFKB1A*). In addition, AgNP exposure increased the expression of *HSPA1A* mRNA and the corresponding stress-induced Hsp72 protein. Up-regulation of Hsp72 by AgNP can suppress *M*.*tb*-induced NF-κB activation and host immune responses. The observed ability of AgNP to modulate infectious pathogen-induced immune responses has important public health implications.

## Introduction

Increased use of nanoparticles (NP) as additives in consumer products may cause frequent human exposures to NP. NP are single particles with diameters between 1 and 100 nm and have unique characteristics that differ from the same material in bulk form [1]. Due to the large specific surface areas, NP are potentially more reactive upon interaction with biological systems than larger particles [2–4]. NP that are taken up by innate immune cells (such as macrophages) can activate or suppress immune system functions [5] with potential consequences for the development of cancer, inflammatory or autoimmune diseases and alterations of host immune responses to pathogens [6]. Although the innate immune system is critical in sensing exposure to NP and functions as a first line of defense against infectious pathogens, only a few studies have assessed the interactions of NP with the human immune system [7–9].

Because of their antibacterial properties [10], silver (Ag) NP (AgNP) are among the most widely used NP in consumer products such as textiles, disinfectant sprays, antibacterial ointments, bandages and medical devices such as orthopedic implants. AgNP are also considered as vehicles for drug delivery and tissue-targeting *e*.*g*. through intravenous application [11,12]. Human exposure to AgNP may occur by inhalation *via* the respiratory tract, by adsorption through the skin, by ingestion through the digestive tract, by implantation, insertion of medical devices or occupationally in the nano-Ag manufacturing industry [13]. Differential effects of AgNP size and surface capping have recently been reported on cellular viability and inflammation and injury in murine lungs [14]. Physicochemical properties of AgNP such as surface charge are largely determined by the capping agents used to prevent NP aggregation. AgNP that are stabilized with weak capping agents such as citrate, which is weakly bound to the Ag core and stabilizes by charge repulsion, tend to aggregate more than those stabilized with strong capping agents such as PVP, which is strongly bound to the Ag core and sterically stabilizes AgNP [15–17].

A recent study indicated that AgNP exposures alter the population of intestinal microbiota and gut-associated immune responses in rats [18]. Anti-inflammatory effects of AgNP capped with polyvinlypyrrolidone (PVP) have also been reported in *Chlamydia trachomatis* infected mouse J774 macrophages [19]. However, the effects of AgNP on antibacterial human host immune cell responses have not been systematically examined.

In the current study, we examined the effects of AgNP with different sizes (diameters 20 or 110 nm) and surface modifications (citrate or PVP-coated) on innate immune responses of human monocyte-derived macrophages (MDM) to infection with *Mycobacterium tuberculosis* (*M*.*tb*). *M*.*tb* causes tuberculosis (TB), a disease of major global health impact, and asymptomatically infects a third of the world population [20]. During natural infection, following inhalation, *M*.*tb* is taken up by phagocytic cells including alveolar macrophages in the respiratory tract with potential outcomes such as abrogation of the infection, latency of infection, progression to active disease or development of reactivation disease years after the primary infection [21]. Epidemiological studies have shown that exposures to cigarette smoke and indoor pollution [22–25] increase rates of TB development in endemic settings. *In vitro* studies have shown that cigarette smoke [26] and indoor air pollution particles [27] affect alveolar macrophages and MDM functions in response to *M*.*tb*. We have shown that exposure of immune cells to diesel exhaust particles (DEP) and air pollution particulate matter adversely affects human antimycobacterial immune mechanisms [28,29]. With this background, we hypothesized that exposure to AgNP may alter human host immune responses to *M*.*tb*. To test this hypothesis we examined the effects of four types of AgNP (Ag20-citrate, Ag110-citrate, Ag20-PVP and Ag110-PVP) on innate *M*.*tb*-specific responses of human MDM, which are widely used to study host immune responses to *M*.*tb* [30,31]. We demonstrate that AgNP suppress innate responses of MDM in response to *M*.*tb* infection including the production of IL-1β. We propose that the upregulation of Hsp72 expression in MDM exposed to AgNP is important for AgNP-induced inhibition of *M*.*tb*-induced host immune responses.

## Materials and Methods

### Ethics statement

Study protocol, consent procedures, and recruitment process were approved by the Institutional Review Board of the legacy University of Medicine and Dentistry of New Jersey now Rutgers University under protocol number 0220100112. Written informed consent was obtained from all study subjects on annually IRB-approved, dated and signed consent forms that explained in detail the purpose of the study, the procedures, and the entirely voluntary character of the participation prior to performing any procedures.

### Generation of MDM

Peripheral whole heparinized blood was collected by venipuncture from healthy men and women at Rutgers University (study protocol approved by the Institutional Review Board of the legacy University of Medicine and Dentistry of New Jersey now Rutgers University under protocol number 0220100112). Peripheral blood mononuclear cells (PBMC) were prepared from whole heparinized venous blood by Ficoll gradient centrifugation [32] as described previously [28]. PBMC (5 x 10^6^) were plated in 6-well plates (BD Falcon, Franklin Lakes, NJ) in 3 mL of complete culture medium per well and incubated at 37°C in a humidified 5% CO_2_ environment for two hours. Following incubation, nonadherent cells were removed by washing twice with RPMI1640 supplemented with Penicillin/Streptomycin/Glutamine (P/S/G). Plastic adherent cells (monocytes) were cultured at 37°C in humidified 5% CO_2_ environment for 7 days, which allows for differentiation into macrophages (MDM). Expression of macrophage-specific surface markers CD11b, CD11c, CD14, CD16, CD163 and HLA-DR on MDM were assessed by flow cytometry with a Gallios Flow Cytometer (Beckman Coulter, Miami, FL) and data analyzed with Kaluza Analysis Software (Beckman Coulter). MDM revealed a typical, earlier described [33–35], phenotype (S1 Table, S1 Fig).

### AgNP characterization

The NCNHIR (NIEHS Centers for Nanotechnology Health Implications Research) consortium provided the AgNP that had been manufactured by nanoComposix, Inc (San Diego, CA) for this study. All four AgNP had a 5–7 nm gold core that served as the nucleation centers during the manufacture of the AgNP via base-catalyzed reduction of silver nitrate. Physicochemical properties of the AgNP were characterized at the Nanotechnology Characterization Laboratory (NCL, National Cancer Institute at Frederick, SAIC-Frederick, Inc. Frederick, MD 21702, http://ncl.cancer.gov) under NIEHS-NCL Agreement NCL-NIEHS201111A. Additional analyses of the morphology and primary size distribution of the AgNP were performed at Imperial College London, using a JEOL 2000 transmission electron microscope (TEM), operated at an accelerating voltage of 200 kV. AgNP suspensions were sonicated for 10 seconds, a single drop of the suspension was deposited onto a 300 Cu Mesh grid with holey carbon support film, and left to dry under vacuum for TEM imaging and analysis. To assess the effects of pH on the stability of the AgNP, the AgNP were incubated in pH 7, sodium perchlorate (NaClO_4_)/perchloric acid (HClO_4;_ Sigma-Aldrich) solution at pH 7, 37°C for 24 h, in the dark [36]. This solution was used to minimize the impact of anions on the stability of the AgNP. To correspond approximately to the pH of extracellular media pH 7 was chosen. Before TEM imaging and analysis, the AgNP in solution, were washed three times in DI-H_2_O using a centrifugation and re-dispersion process, to remove excess salts. A full description and discussion of these methods and comparison of TEM results to *in situ* small angle x-ray scattering analysis, including discussion of possible drying artefacts, are provided elsewhere [36].

### Stability of AgNP: dissolution kinetics

Inductively coupled plasma-optical emission spectroscopy (ICP-OES) was employed to analyze aliquots of Ag solutions at various time points from 1 hour up to 3 days (72 hours). The amount of dissolved Ag was determined at pH 5 and 7, matching approximately the pH found in the lysosomes and extracellular media, respectively. Non-interacting buffers were used to minimize the impact of anions on the stability of the AgNP. Each of the AgNP suspensions were incubated in a temperature controller at 37°C followed by centrifugation with 2kDa (< 4 nm) filter tubes (Sartorius Stedim VIVACON 500) at 13,000 rpm to separate any AgNP from the solution. The concentration of released Ag^+^ ions was assessed after the AgNP had been removed at 1, 6, 24 and 72 hours (n = 3). In addition, deionized water (no AgNP) as well as supernatant, after the removal of the AgNP, were analyzed in control experiments ensuring that residual AgNP were removed during centrifugation and filtering.

### Preparation of AgNP and *M*.*tb* for *in vitro* exposure and infection

For *in vitro* MDM exposure experiments, AgNP samples were diluted in complete cell culture medium without antibiotics (RPMI1640 supplemented with 10% pooled human AB serum) followed by sonication in a Branson 3510 water bath sonicator for 2 minutes prior to addition to the cell cultures. To assess the potential contribution of the stabilizers PVP10 (used to stabilize Ag20) and PVP40 (used to stabilize Ag110) to AgNP-induced cell toxicity or alterations of immune responses, mass equivalents of PVP10 and PVP40 within the 5, 10, 20 and 50 μg/mL exposure concentrations of Ag20-PVP and Ag110-PVP samples were calculated. Calculations of the PVP10 and PVP40 equivalents based on data supplied by NCL according to which PVP10 and PVP40 concentrations were 33.3 and 62.4 μg per 1 mg of Ag in Ag20-PVP and Ag110-PVP, respectively.

MDM were infected with avirulent *M*.*tb* strain (H37Ra, ATCC catalog number 25177) at a multiplicity of infection (MOI) of ten bacilli (MOI 10) per cell.

### MTS assay

Cell viability was assessed by Cell Titer 96 Aqueous One Solution Cell Proliferation Assay [MTS, (3-(4,5-dimethylthiazol-2-yl)-5-(3-carboxymethoxyphenyl)-2-(4-sulfophenyl)-2H-tetrazolium), Promega,Madison WI] as per the manufacturer’s protocol. Briefly, MDM (44,000/well) were incubated at 37°C in a humidified CO_2_ environment for 3, 6 and 24 hours in presence of 0 (cells alone, negative control), 5, 10, 20 and 50 μg/mL of AgNP (Ag20-citrate, Ag20-PVP, Ag110-citrate, Ag110-PVP) and PVP10 and PVP40 equivalents corresponding to the 5, 10, 20 and 50 μg/mL concentrations of Ag20-PVP and Ag110-PVP. Absorbance was recorded at 493 nm with an ELISA reader (ThermoScientific Multiskan FC, Finland). Percentages of viable cells were calculated as ratios of ODs of NP-exposed MDM (after background subtraction) to the ODs of unexposed MDM (after background subtraction) x 100.

### Lactate dehydrogenase (LDH) assay

Cell culture supernatants (50 μL) were collected following MDM exposures to AgNP and PVP10 and PVP40 controls and transferred into 96-well assay plates. Fifty μL of substrate (CytoTox 96 Non-radioactive cytotoxicity Assay, Promega, Madison, WI) was then added per well. Following incubation at room temperature for 30 minutes in the dark, stop solution (50 μL) was added to each well and absorbance recorded at 493 nm as described above. Cellular toxicity was defined as percent (%) LDH leakage from cells calculated from the ratios of ODs of NP-exposed MDM (after background subtraction) to the ODs of unexposed MDM (after background subtraction) x 100.

### Quantitative RT-PCR to determine cytokine mRNA abundance

The following primer sets were used for qRT-PCR, *TNFA* forward: GTGCTTGTTCCTCAGCCTCTT, *TNFA* reverse: ATGGGCTACAGGCTTGTCATC; *IL1B* forward: GAAGCTGATGGCCCTAAACAG, *IL1B* reverse: AGCATCTTCCTCAGCTTGTCC; *IL8* forward: ACTGAGAGT GAT TGA GAG TGG AC, *IL8* reverse: AAC CCTCTGCACCCAGTTTTC; *IL10* forward: ACCTGCCTAAGATGCTTCCAG, *IL10* reverse: CTGGGTCTTGGTTCTCAGCTT; *HSPA1* forward: AAGTACAAAGCGGAGGACGAG, *HSPA1* reverse: CCACGAGATGACCTCTTGACA. For each of the mRNAs, fold-changes relative to unexposed MDM were calculated as described previously [28].

### Pathway-specific qRT-PCR arrays

Human Toll-like Receptor (TLR) signaling pathway-specific RT^2^-Profiler arrays (Cat. No. PAHS 018E, Qiagen Sciences, MD) were used to screen for mRNA expression in AgNP and *M*.*tb*-exposed and unexposed MDM [28]. Levels of cDNA were calculated by the relative quantitation method (ΔΔC_t_ method) from the PCR array data using analysis software accessed from http://sabiosciences.com/pcrarraydataanalysis.php. Statistical differences in fold-mRNA expression levels between exposed and unexposed cells were calculated using the same software.

### ELISA for IL-1β detection

Following plastic adherence for 7 days (as described above), MDM were treated with 5 mM EDTA/PBS for 5 minutes and then gently scraped from six-well plates and subsequently plated into 96-well plates. On the next day, cells were exposed to 0, 1 and 10 μg/mL of NP with simultaneous infection with *M*.*tb* at MOI 10. Culture supernatants from *M*.*tb*-infected and uninfected MDMs were collected at 6 and 8 hours, centrifuged for 5 minutes at 5,000 rpm to remove particles and bacteria, and assessed for IL-1ß protein content by ELISA following the manufacturer’s protocol (R&D Systems, Minneapolis, MN). Absorbance was recorded at 450 and 570 nm with an ELISA reader (ThermoScientific Multiskan FC, Finland).

### Flow cytometry for Hsp72 detection

MDM were washed with flow cytometry staining buffer and fixed in IC (intracellular) fixation buffer (eBiosciences, Inc. San Diago, CA). Fixed cells were permeabilized with 1x permeabilization buffer (eBiosciences) followed by staining with FITC conjugated anti-Hsp72 (cat. No. ADI-SPA-810FI-D, Enzo Life sciences, Farmingdale, NY) or mouse IgG1 isotype control (cat. No. ADI-SAB-600FI-050, Enzo Life sciences) for 20 minutes at room temperature in the dark. After washing, the expression of Hsp72 in MDM was evaluated by flow cytometry with a Gallios Flow Cytometer (Beckman Coulter, Miami, FL) and analyzed with Kaluza Analysis Software (Beckman Coulter).

### Colony forming unit (CFU) assay

To evaluate direct, AgNP-mediated antibacterial effects, *M*.*tb* was grown in cell culture media in the presence or absence (control) of Ag20-citrate, Ag20-PVP, Ag110-ctrate, and Ag110-PVP. Ten μL of *M*.*tb* stock (CFU 1 x 10^7^/mL) were diluted to 1 mL with cell culture media (RPMI1640 + 10% PHS) and incubated at 37°C with rotation on a revolver (Labnet International, Inc., Edison, NJ) in the presence of 0, 10 and 25 μg/mL of AgNP and 15.7 and 39.25 μg/mL of AgNO_3_ for 24 hours. AgNO_3_ concentrations corresponded to the total amounts of Ag^+^ ions present in 10 and 25 μg/mL of AgNP, respectively. Ten μL of the culture media samples were removed at 24 hours and serially 10-fold diluted in cell culture media without antibiotics (RPMI1640 + 10% PHS) and plated in triplicate onto 7H10 agar plates and incubated for 21 days at 37°C [37]. *M*.*tb* colonies (CFU) were counted using a stereo microscope at 40x magnification (Diagger Lab equipments, Vernon Hills, IL), and plotted as a function of the AgNP concentration. *M*.*tb* grown in NP-free culture media served as a control.

### *M*.*tb* uptake/internalization

Enriched CD14^+^CD3^-^ PBMC were generated by negative selection through immunodepletion of non-monocytes as described previously [28]. For generation of MDM, enriched monocytes were plated onto 8-chamber (5x 10^5^/chamber) culture slides (BD-Falcon, Cat. No. 354118) and allowed to differentiate for 7 days. Culture media were changed every 2–3 days during the 7-day incubation period. MDM were infected with *M*.*tb* at MOI 10 in presence of Ag20-citrate (10 μg/mL) or Ag110-citrate (10 μg/mL) for 2 and 4 hours. Following incubation, extracellular bacteria were removed by extensive washing with RPMI 1640 and slides were stained with Kinyoun Staining fluid as described previously [28]. Proportions of MDM harboring phagocytosed bacilli (stained in red) were assessed by oil immersion bright field microscopy (100X, Zeiss Primo Star) in a total of 300 MDM for each experimental condition.

To determine the number of *M*.*tb* that had been internalized within individual MDM, MDM (2 x 10^5^ monocyte/well) were grown in 96-well culture dishes, infected with *M*.*tb* as described above and washed after 4 hours to remove extracellular bacteria. MDM were then lysed by addition of 0.1% sodium dodecyl sulfate (SDS) in 7H9 Milddlebrook medium for 10 min at room temperature after which SDS was neutralized with 20% Bovine Serum Albumin (BSA). Lysed cells were serially diluted with 7H9 medium and plated out in triplicate onto agar plates (Middlebrook 7H10 agar) and incubated for 21 days at 37°C. CFU numbers were then determined as described above.

### Statistical analysis

Mixed linear models that include a random effect for experiments, no fixed intercept and an estimated common variance across doses were used to estimate the effect of dose within each condition on the logarithm of the fold-change. Previous examination of summary statistics demonstrated a relatively constant variance of the log-transformed fold-changes. Thus, with the small sample sizes (n = 4) for each dose, this approach would potentially give the most stable variance estimates. Wald tests examined whether the log-fold changes were significantly different than zero or, equivalently, whether the fold-changes were significantly different from one. Fold-changes of cytokine expression under exposure at doses of 1, 10 or 25 μg/mL relative to expression at no exposure were calculated within each stimulus-by-*M*.*tb* exposure combination. To examine AgNP dose effects on percent viability and LDH leakage, mixed linear models similar to those described above were used. Analyses were stratified by stimulus as well as hour. The two-tailed, unpaired Student’s t-test was used to examine (i) the effects of AgNP, carbon black and Ag^+^ ions on IL-1β expression, (ii) the effect of AgNP on *M*.*tb* phagocytosis/internalization, and (iii) *IL1B* and *HSPA1* expression.

## Results

### Characterization of AgNP

**Fig 1:**
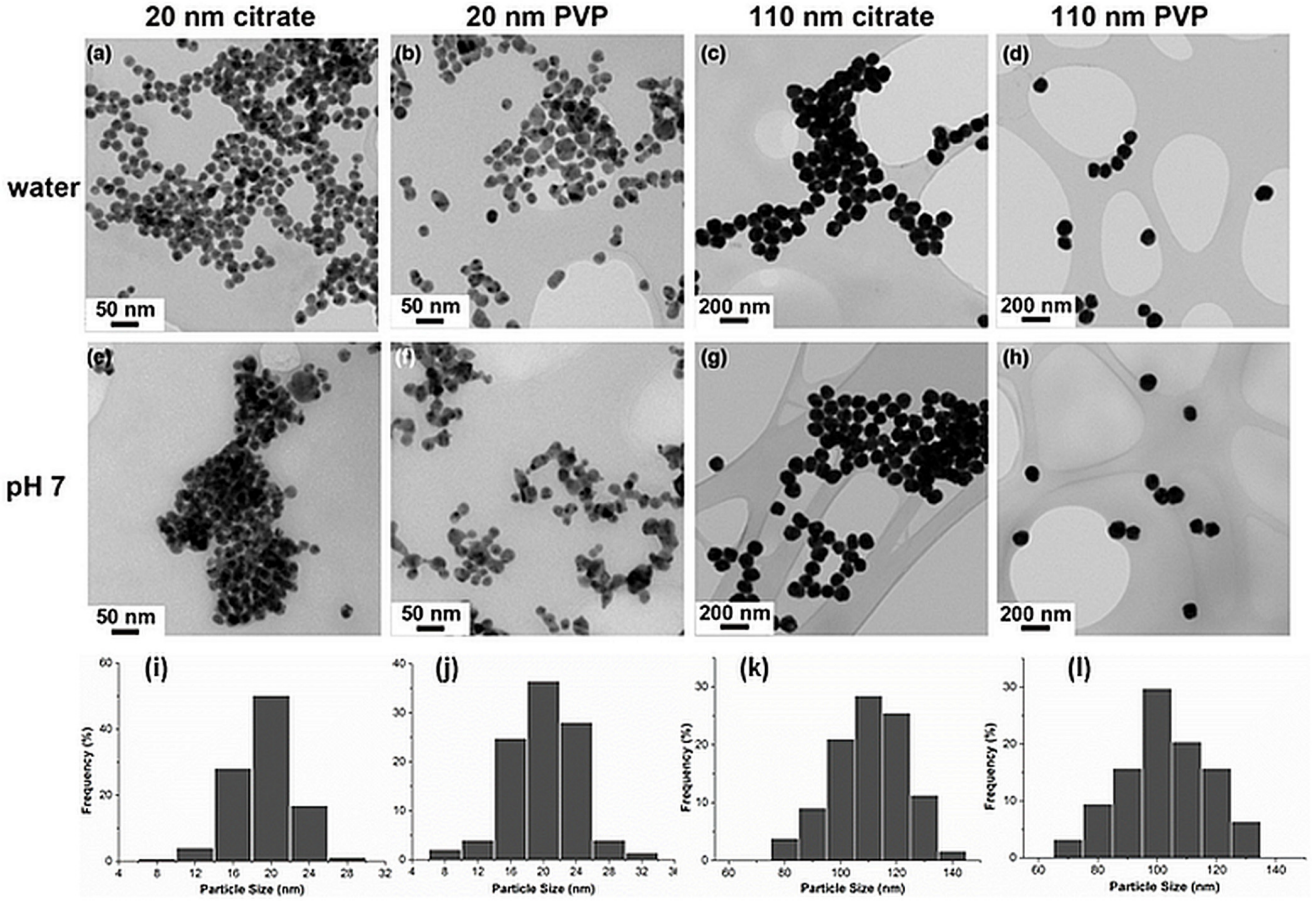
Characterization of AgNP by TEM. TEM images of citrate compared with PVP stabilized AgNPs (20 nm and 110 nm) in (**a**—**d**) DI water and (**e**—**h**) incubated in pH 7 perchlorate buffer solution at 37°C for 24 hours (magnification at 20 x); (**i**—**l**) show the corresponding (i, a and e; j, b and f; k, c and g; l, d and h) size distribution histograms of AgNP in DI water (n = 200 repeat measurements).

Physicochemical properties of AgNP are crucial determinants of their bioreactivities. Information on size (determined by dynamic light scattering after diluting AgNP 10-fold in 2 mM NaCl to measure the hydrodynamic diameter), charge (determined by Zeta potential measurement at pH 7), and endotoxin concentration (analyzed by limulus amebocyte lysate assay) of the AgNP (supplied by NCNHIR consortium) was provided by the NCL and is listed in Table 1. Endotoxin levels in AgNP were low reducing the likelihood that any observed AgNP induced immune modulations were endotoxin-induced. The primary size distribution of the citrate and PVP-stabilized Ag20 and Ag110 in water (Fig 1A–1D) and pH7 solution (Fig 1E–1H) was compared by TEM performed at Imperial College, London. The size distributions of the different AgNP preparations studied here indicate that the diameters of the majority of Ag20 and Ag110 are ˃16 nm (Fig 1I and 1J) and ˃80 nm (Fig 1K and 1L), respectively. Assessments confirmed that physicochemical properties of the as–received AgNP (dispersed in DI water) were consistent with the specifications provided by NCL. Additional characteristics of AgNP with gold core are discussed elsewhere by Botelho *et al*.[38].

**Table 1:** Physicochemical properties of AgNP.

Samples	Diameter (nm)of >94% of particles	Zeta potential (mV)	Endotoxin conc.(EU/ml)
20 nm, Citrate-stabilized Ag	20.9 ± 2.1	-44.3 ± 1.3	<0.05
20 nm, PVP-stabilized Ag	20.9 ± 4.9	-38.2 ± 1.6	2.2
110 nm, Citrate-stabilized Ag	114.9 ± 8.5	-45.2 ± 0.4	<0.05
110 nm, PVP-stabilized Ag	117.4 ± 8.6	-31.6 ± 2.2	<0.05

**Source**: Characterization Data for Silver Nanomaterials, prepared by NCL, NCL-NIEHS201111A. AgNP size was determined by dynamic light scattering after diluting AgNP 10-fold in 2 mM NaCl to measure the hydrodynamic radius. Charge and endotoxin contamination were determined by Zeta potential measurement at pH 7 and limulus amebocyte lysate assay, respectively.

**Fig 2:**
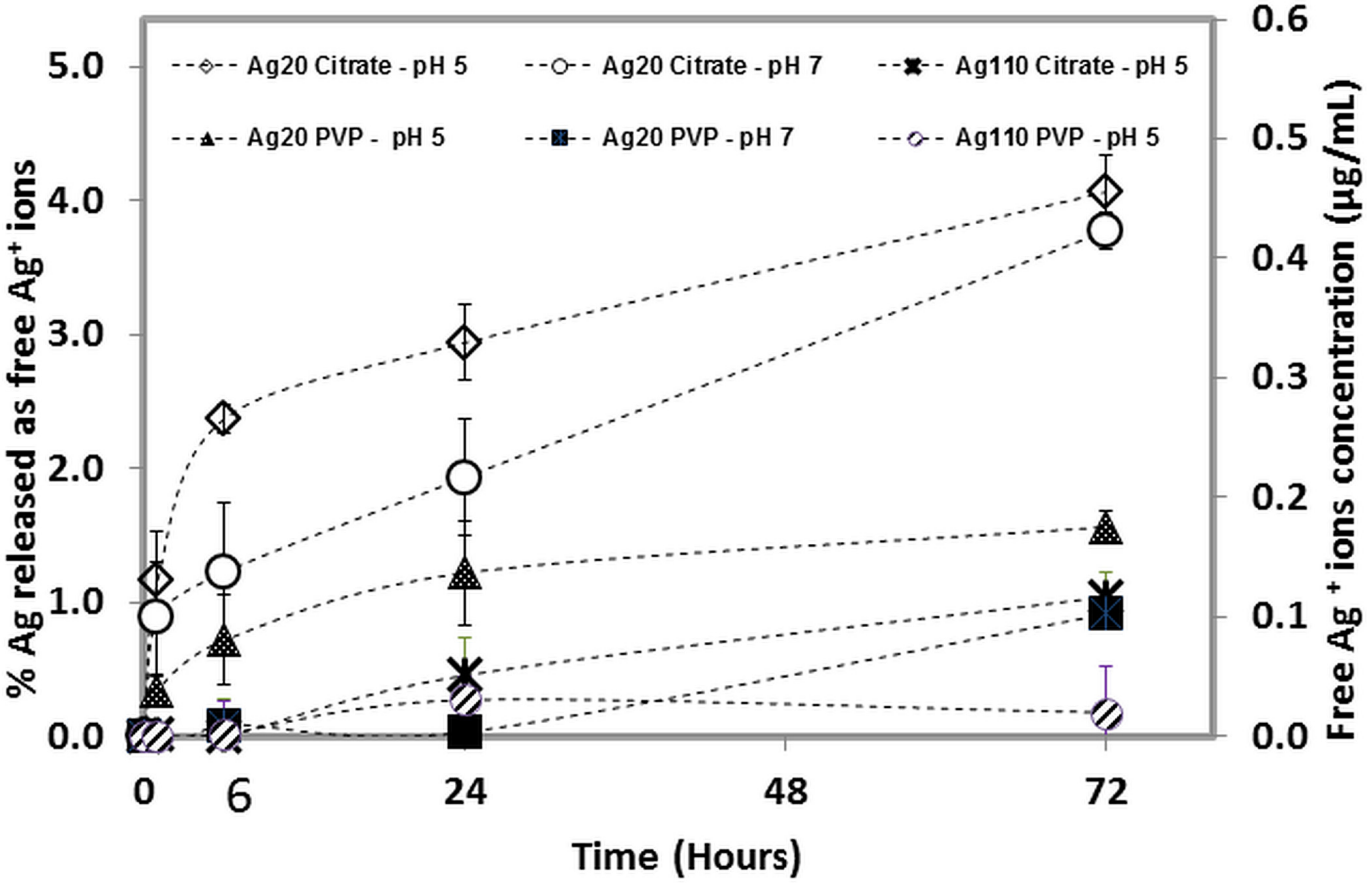
Dissolution kinetics of AgNP. Ag^+^ ion release from citrate and PVP-capped Ag20 and Ag110 NP incubated in perchlorate acid/ perchlorate buffer solutions (pH 5, and 7).

Our previous work [36] showed that dissolved Ag^+^ ions reprecipitate as insoluble Ag salts (*e*.*g*. Ag_2_O, AgCl and Ag_2_S) in the cell culture medium (RPMI and 10% pooled human serum) used in this study. For this reason, we decided not to measure the amount of ionic Ag^+^ in the cell culture medium as it was expected that the amount of free Ag^+^ ions in this medium would be below the detection limit of ICP, and not representative of the actual Ag^+^ ion-release. Therefore, ICP was employed to measure the dissolution rates of the AgNP in inorganic buffers at both pH 5 & 7. All AgNP showed a higher rate of dissolution at pH 5 than at pH 7 (Fig 2). Except for Ag20-citrate, all other AgNP released less than 2% Ag^+^ ions into the solution over a three-day incubation period (72 h). Furthermore, Ag^+^ ion levels were undetectable in either Ag110 citrate or Ag110 PVP in pH 7 buffer solutions, indicating that AgNP dissolution was negligible. Other studies have reported a 5 to 15% dissolution from citrate-stabilized particles [39]. Discrepencies between these studies and our findings are probably due to differences in methodology, dissolution medium, and separation techniques used to assess Ag^+^ ion dissolution. As seen in Fig 2, Ag20-citrate showed greater dissolution than the other AgNP at all time points at both pH 5 and 7 displaying >4% Ag^+^ ion release into the solution after 72 hours of incubation. In general the amounts of Ag^+^ ion release were very low (<4% of total available Ag) in the non-interacting solutions for all AgNP formats (4 hours) tested in this study.

### Cytotoxic effects of AgNP on human MDM

**Fig 3:**
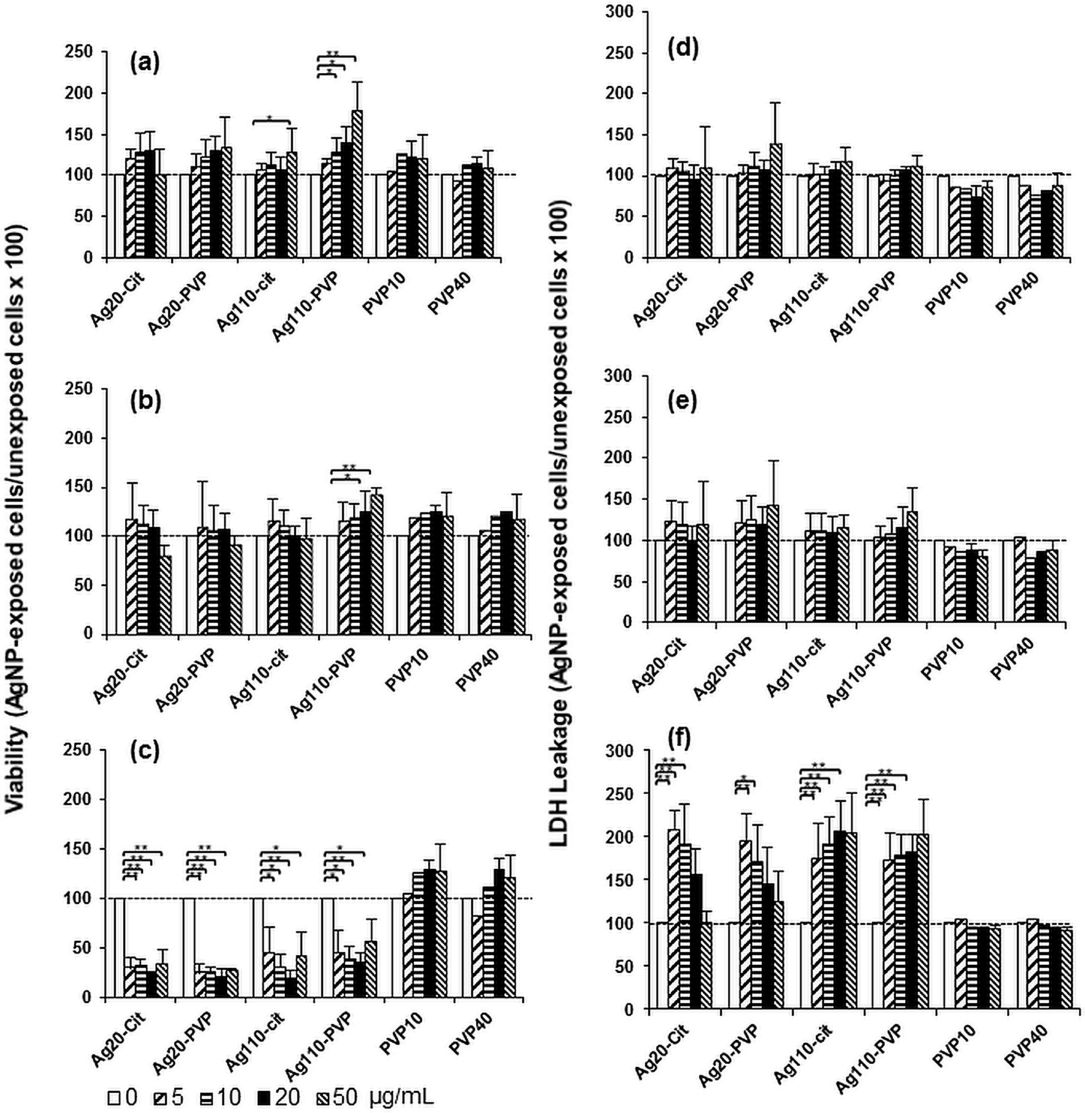
Cytotoxic effects of AgNP in MDM. MDM were exposed to 5, 10, 25 and 50 μg/mL of Ag20-cit, Ag20-PVP, Ag110-citrate, Ag110-PVP and the stabilizers PVP10 (0.2, 0.3, 0.7 and 1.7 μg/mL) and PVP40 (0.3, 0.6, 1.3 and 3.1 μg/mL) for 3, 6 and 24 hours at 37°C in a humidified 5% CO_2_ environment. MDM cultured in complete culture media without NP exposure (0) were used as unexposed controls. Cell viability (panels **a**, **b** and **c**) and cytotoxicity proportional to LDH leakage (panels **d**, **e** and **f**) were measured by MTS and LDH assays, respectively. Percentages of viable cells were calculated as ratios of ODs of NP-exposed MDM (after the subtraction of background) to the ODs of unexposed MDM (after background subtraction) x 100. Toxicity was defined as percent (%) LDH leakage from cells calculated from the ratios of ODs of NP-exposed MDM (after background subtraction) to the ODs of unexposed MDM (after background subtraction) x 100. Each data point represents the mean ± SD from three independent experiments. Statistical significance relative to unexposed control MDM are shown as * (*p*<0.05) or ** (*p*<0.01). Horizontal dashed lines represent the level of viable cells (**a**, **b**, and **c**) or LDH (**d**, **e**, and **f**) leakage in unexposed MDMs.

Because AgNP are reported to be cytotoxic to a variety of human cells [40–42], we first examined whether AgNP exposures would affect the viability of MDM. MDM were exposed to 0 (unexposed control), 5, 10, 20, 50 μg/mL of AgNP or the stabilizers PVP10 or PVP40 alone for periods of 3, 6 and 24 hours. The AgNP dose ranges were similar to those (6.25, 12.5, 25 and 50 μg/mL) recommended by the NCNHIR consortium and used previously in *in vitro* studies by others [14]. An increase in metabolic activity (*p*<0.05) was observed in MDM exposed to 50 μg/mL of Ag110-citrate and to 10, 20 and 50 μg/mL of Ag110-PVP for 3 hours (Fig 3A) and 20 and 50 μg/mL of Ag110-PVP for 6 hours (Fig 3B) relative to unexposed control MDM as determined by MTS assays. A dramatic reduction in the proportion of viable cells was observed in MDM exposed to all four AgNP for 24 hours starting at concentrations as low as 5 μg/mL (Fig 3C). Neither of the capping agents (PVP10and, PVP40) had statistically significant effects on the viability of MDM during 3, 6 or 24 hour cell exposures (Fig 3A–3C). The significance of the increase in metabolic activity relative to control cells is not clear. MTS assays rely on a mitochondrial reductase to convert the tetrazole to formazan, which is dependent on the number of viable cells. As the exposure of MDM to AgNP may result in increased enzymatic activity without actually having an effect on cell number or cell viability we also performed LDH cytotoxicity assays (see below) to complement the results of the MTS assays.

LDH leakage from the cells, a measure of cytotoxicity, was not significantly increased following MDM exposure to AgNP for periods of 3 and 6 hours compared to unexposed control cells (Fig 3D and 3E). Nevertheless, levels of LDH release nearly doubled in MDM exposed to each of the four AgNP types for 24 hours relative to unexposed MDM (Fig 3F). Interestingly, a gradual decrease of LDH release in MDM exposed to increasing concentrations of Ag20-citrate and Ag20-PVP but not to Ag110-citrate or Ag110-PVP was noted (Fig 3F). This unexpected finding may be explained by interferences of the dark Ag20-citrate and Ag20-PVP suspensions with the OD readings of the LDH concentrations (Fig 3F) in the assay. As expected, LDH release was inversely correlated with the MTS assay measurements of MDM viability after 24 hours of AgNP exposure (compare Fig 3C and 3F).

Taken together, these results indicate that AgNP, but not their stabilizers (PVP10 and PVP40), were cytotoxic for MDM during extended exposure periods (24 hours) while shorter exposure periods (3 or 6 hours) did not induce significant toxicity. Unlike studies with cultured cell lines that show greater toxicity upon exposure to smaller AgNP [14], no differences in size and/or stabilizer-associated AgNP toxicity were observed in primary MDM in the current study.

### Cytokine expression in *M*.*tb*-infected MDM in response to AgNP exposure

**Fig 4:**
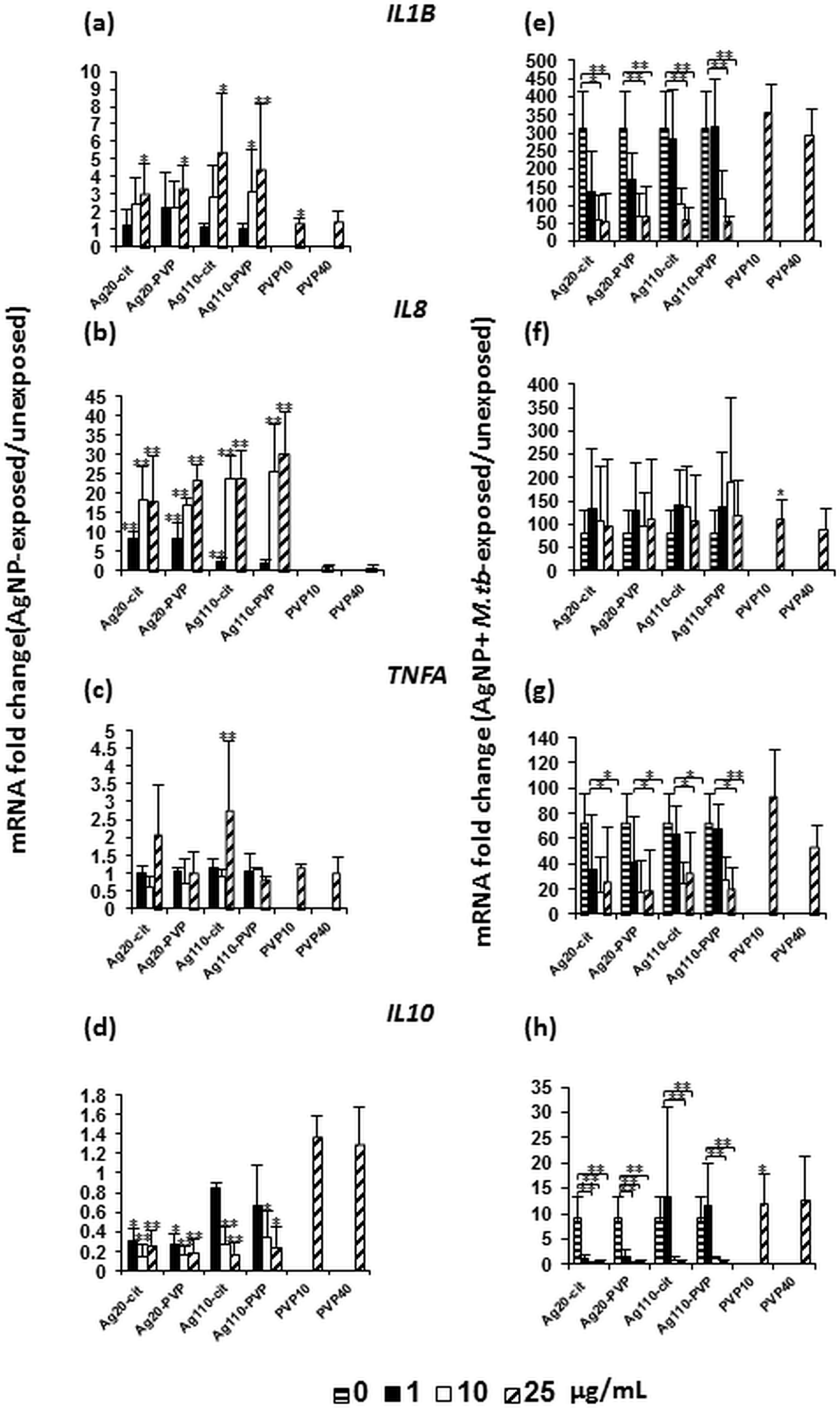
Effect of AgNP on cytokine mRNA expression by MDM in the absence and presence of *M*.*tb*. MDM from four healthy human blood donors were exposed to 0, 1, 10 and 25 μg/mL of Ag20-citrate, Ag20-PVP, Ag110-citrate, Ag110-PVP and 1.7 μg/mL PVP10 and 3.1 μg/mL PVP40 in the absence (**a**, **b**, **c** and **d**) or presence (**e**, **f**, **g**, and **h**) of *M*.*tb* (MOI 10) for 4 hours at 37°C in a humidified 5% CO_2_ environment. Following incubation, RNA was extracted and the abundance of mRNA encoding *IL1B*, *IL8*, *TNFA* and *IL10* examined by qRT-PCR using gene-specific primer sets as described [28]. For primer sequences see Materials and Methods. Results are shown as fold-changes relative to controls (MDM in culture media without AgNP (0 μg/mL) in **a-h**. In panels **e-h**, 0 μg/mL data points represent MDM exposed to *M*.*tb* MOI 10 in absence of AgNP. Each data point (Y-axis) represents mean fold-changes ± SD from four independent experiments. Statistically significant changes relative to unexposed MDM are marked by * (*p* < 0.05) or ** (*p* <0.01).

Since macrophages are capable of phagocytosing *M*.*tb* and act as primary mediators of antimycobacterial immune responses, we set out to assess AgNP effects on inflammatory responses of MDM to *M*.*tb*. Because of their important roles in protective antimycobacterial immunity, IL-1β, IL-8, TNF-α and IL-10 were assessed as macrophage products in this study. MDM were first exposed to AgNP at concentrations of 0, 1, 10 and 25 μg/mL for 4 hours, a time period during which no significant AgNP-mediated cytotoxicity on MDM had been observed (see section on cytotoxic effects above). The abundance of mRNAs encoding *IL1B* (Fig 4A) and *TNFA* (Fig 4C) changed moderately and significantly (*p*<0.05) at the 25 μg/mL concentration only. Interestingly, a dose-dependent increase in the expression of mRNA encoding *IL8* (Fig 4B) was observed with increasing concentrations of all four AgNP types. In contrast, the abundance of mRNA encoding *IL10* (Fig 4D) was reduced in the presence of AgNP relative to unexposed MDM. Taken together, these data indicate that inflammatory cytokine responses were increased in the presence of AgNP in a concentration-dependent manner.

Next, MDM were exposed to AgNP and simultaneously infected with *M*.*tb*. Expression of mRNAs encoding *IL1B*, *TNFA* and *IL10* was significantly (*p*<0.05) reduced in AgNP-exposed and *M*.*tb* infected MDM compared to unexposed MDM infected with *M*.*tb* only (0 μg/mL AgNP) (Fig 4E, 4G and 4H). No statistically significant effects on mRNA encoding *IL8* were observed in MDM exposed to any of the four AgNP types and infected with *M*.*tb* relative to MDM infected with *M*.*tb* alone (Fig 4F). It is worth noting that AgNP-induced inhibitory effects on *IL10* mRNA expression in the presence of *M*.*tb* were observed starting at lower doses with Ag20 (*p* <0.05, 1 μg/mL) than with Ag110 (*p* <0.05, 10 μg/mL) (Fig 4H). Exposure of MDM to *M*.*tb* and the stabilizers PVP10 or PVP40 (in equivalent amounts present in 25 μg/mL of Ag20-PVP and Ag110-PVP), respectively had no significant effects on the expression of *IL1B*, and *TNFA* (compare with 0 μg/mL AgNP, Fig 4E and 4G), indicating that the observed AgNP-induced suppressive effects on *M*.*tb*-induced *IL1B* and *TNFA* mRNA expression were specific to AgNP and not to interferences of the NP stabilizers with the cytokine mRNA expression in MDM.

**Fig 5:**
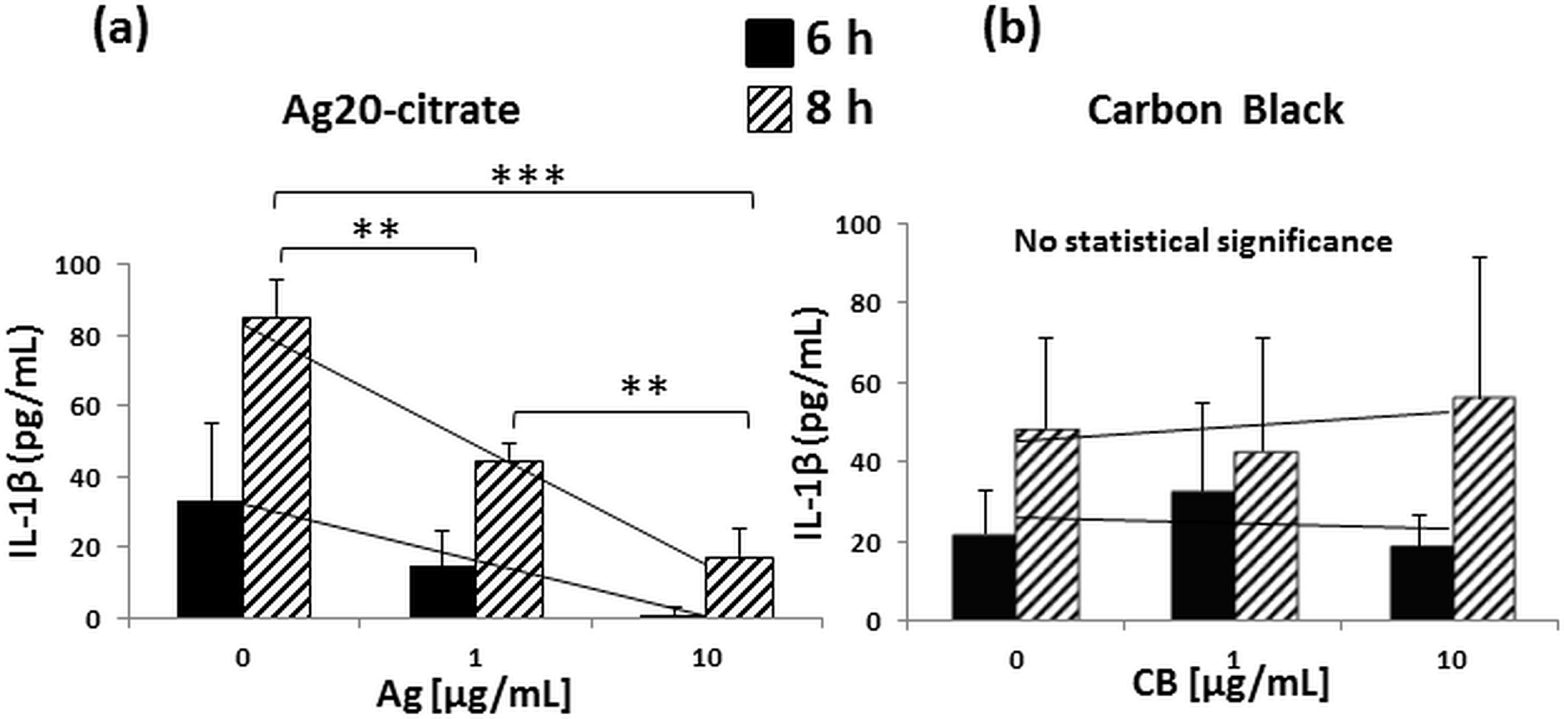
Effect of AgNP and CB on IL-1β production. After differentiation for 7 days, MDM were harvested, counted, and plated at concentrations of 0.7–1 x 10^5^ cells per well into 96-well plates. The next day, MDM were incubated with 0, 1 and 10 μg/mL of Ag20-citrate (a) or CB (b) in the presence of *M*.*tb* at MOI 10 at 37°C and 5% CO_2_ in a humidified environment. MDM cultured in complete media served as negative control. Culture supernatants were collected at 6 and 8 hours and analyzed by IL-1β ELISA as described in Materials and Methods. Each data point (Y-axis) represents pg/mL of IL-1β ± SD from three independent experiments. Statistically significant changes relative to *M*.*tb*-exposed MDM (0 on X-axes) were determined by two-tailed unpaired t-test and marked by ** (*p* < 0.01) or *** (*p* <0.001).

To examine whether changes detected at the level of mRNA were also reflected at the protein level, we focused on IL-1β, a cytokine critical for host resistance to *M*.*tb* [30,43,44]. Assessment of IL-1β expression in MDM exposed to Ag20-citrate and infected with *M*.*tb* for 6 and 8 hours by ELISA revealed that the expression of IL-1β protein was inhibited in the presence of Ag20-citrate in a concentration-dependent manner (Fig 5A) while exposure to carbon black particles (chemically inert control particles in the size range of the Ag20 NP) had no statistically significant effects on *M*.*tb*-induced IL-1β production (Fig 5B). No cytotoxic effects (as per MTS assay) were observed after 8-hour exposures to AgNP or CB (data not shown). Taken together, these data indicate *first* that *M*.*tb*-induced inflammatory cytokine responses are inhibited by AgNP in a concentration-dependent manner and *second* that suppression of the *M*.*tb*-induced proinflammatory cytokine response is specific to AgNP, as carbon black particles failed to inhibit *M*.*tb*-induced IL-1β production.

### Contribution of Ag^+^ ions to the AgNP-mediated suppression of host immune responses to *M*.*tb*

**Fig 6:**
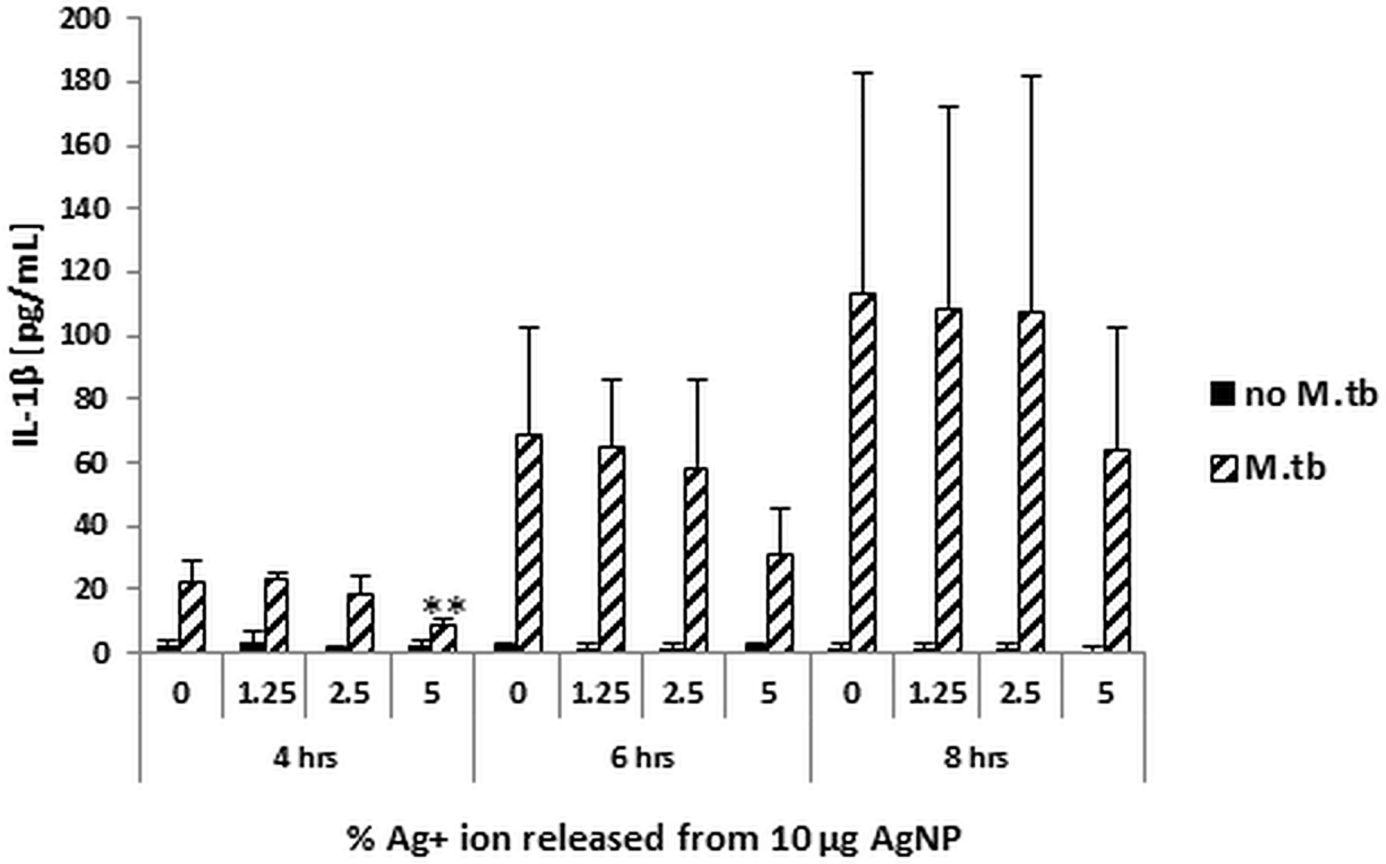
Effect of Ag^+^ ions on *M*.*tb*-induced IL-1β expression. MDM were exposed to AgNO_3_ corresponding to 0.125, 0.25 and 0.5 μg/mL of Ag^+^ ions in the absence or presence of *M*.*tb* at MOI 10. Culture supernatants collected at 4, 6 and 8 hours after infection with *M*.*tb* ± Ag^+^ ion exposures were assessed for IL-1β production. Results are expressed as means ± SD from four independent experiments. Statistically significant changes relative to *M*.*tb*-exposed MDM (0 on X-axes) were determined by two-tailed unpaired t-test and marked by ** (*p* < 0.01).

To determine whether Ag^+^ ions, released as a result of dissolution of AgNP, contribute to AgNP-mediated effects, MDM were exposed to AgNO_3_ corresponding to 0.125, 0.25 and 0.5 μg/mL of Ag^+^ ions in the absence or presence of *M*.*tb*. As shown in Fig 2, 1.25 and 2.5% Ag are released at 6 hours at pH 7 and pH 5, respectively, from 10 μg of Ag20-citrate, which shows the highest rate of dissolution compared to all other AgNP tested in this study. Culture supernatants from MDM collected at 4, 6 and 8 hours were assayed for IL-1β production. Exposure of MDM did not inhibit *M*.*tb*-induced expression of IL-1β in presence of 1.25 or 2.5% Ag^+^ (Fig 6). A consistent decrease in IL-1β expression, although not statistically significant with the exception of the 4-hour time point, was observed at the 5% level of Ag^+^, which reflects a greater amount of dissolved Ag^+^ than that actually found from Ag20-citrate during a 72-hour period (Fig 2). Thus, the AgNP-mediated suppressive effect on *M*.*tb*-induced IL-1β expression is unlikely to be due to the presence of Ag^+^ ions.

### Effect of AgNP on the viability of *M*.*tb*

**Fig 7:**
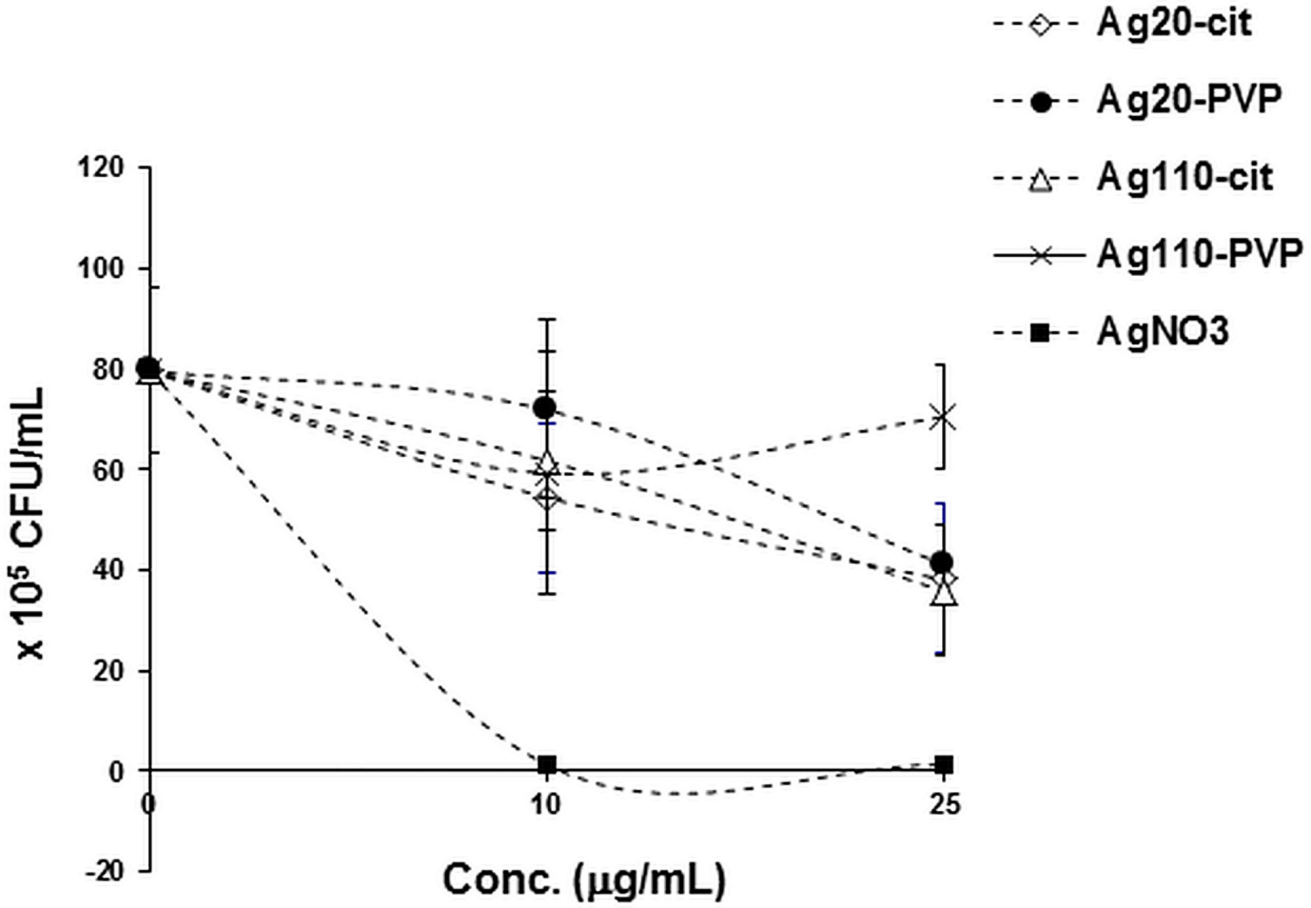
Effect of AgNP on the viability of *M*.*tb*. Ten μl of *M*.*tb* stock suspension (10^7^ CFU/mL) was diluted to 1 mL with cell culture media (RPMI1640 + 10% PHS, reflecting the environment in which AgNP and *M*.*tb* interacted with the MDM *in vitro*) and incubated at 37°C on a rotating shaker in the presence of 0, 10 and 25 μg/mL of AgNP and 15.7 and 39.25 μg/mL of AgNO_3_. The amounts of AgNO_3_ corresponded to the amount of Ag^+^ ions present in 10 and 25 μg/mL of AgNP, respectively. *M*.*tb* culture samples (10 μL) were removed at 24 hours, diluted 10-fold by serial dilution, plated in triplicate onto 7H10 agar plates and incubated for 21 days at 37°C. CFU were determined as described in the Materials and Methods and plotted as a function of AgNP or Ag^+^ ion concentration. Mean values from two independent experiments ± SD are shown.

As live *M*.*tb* induces greater host immune responses in MDM than heat-killed *M*.*tb* [45–47], we examined whether the observed AgNP dose-dependent suppression of *M*.*tb*-induced host responses (Figs 4 and 5) could be related to a direct mycobactericidal effect of AgNP. Because AgNP are known to exhibit antibacterial properties we assessed AgNP effects on *M*.*tb* growth in CFU assays. *M*.*tb* growth was reduced by 10–20% only upon 24-hour exposure of *M*.*tb* to any of the AgNP at the 10 μg/mL concentration (Fig 7) at which AgNP-mediated suppression of *M*.*tb*-induced mRNA and protein expression were observed (Figs 4 and 5). As our AgNP suppression studies were done at 4-hr time points, these data strongly indicate that the observed AgNP-mediated suppression of *M*.*tb-*induced MDM responses resulted from interferences of AgNP with *M*.*tb*-induced host cell signaling pathways and not from direct mycobacteriocidal effects of the AgNP. AgNO_3_ used as a positive control for *M*.*tb* killing (see Material and Methods) completely abrogated *M*.*tb* growth.

### Effect of AgNP on the internalization of *M*.*tb* in MDM

**Fig 8:**
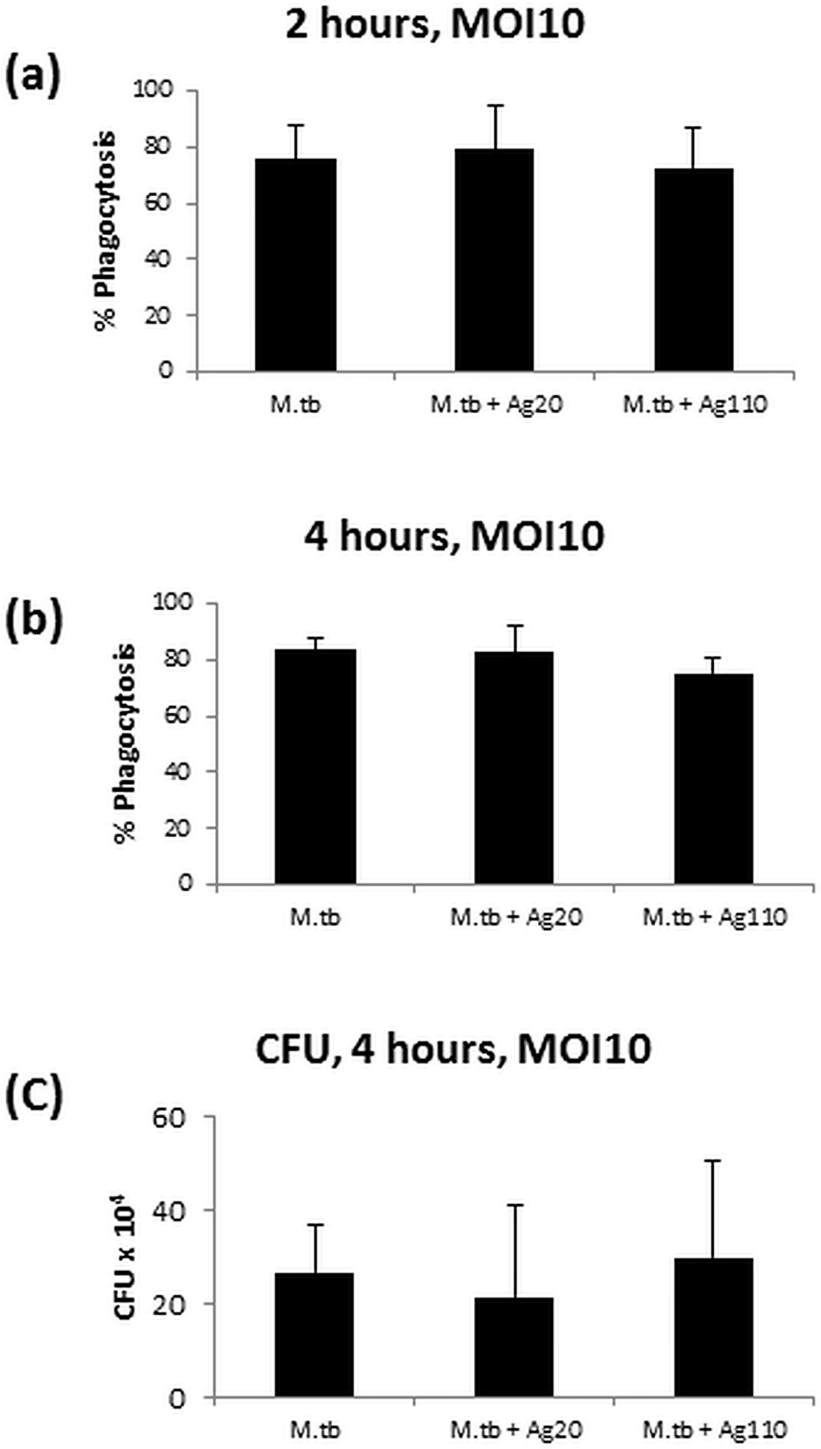
Effect of AgNP on the internalization of *M*.*tb* by MDM. MDM were infected with *M*.*tb* at MOI 10 in the absence or presence of 10 μg/mL of AgNP and the percentage of MDM with internalized *M*.*tb* determined after 2 (panel **a**) and 4 (panel **b**) hours of infection. A total of 300 MDM was examined in each experimental condition as described in Materials and Methods. Results are expressed as mean percentages of MDM with internalized *M*.*tb* ± standard deviations from four independent experiments. To assess the number of internalized *M*.*tb* in MDM, MDM infected with *M*.*tb* (MOI 1) alone, MDM infected with *M*.*tb* and exposed to Ag20-citrate (10 μg/mL) and MDM infected with *M*.*tb* and exposed to Ag110-citrate (10 μg/mL) were lysed following a 4-hour incubation. MDM lysates were then plated onto agar plates and CFU determined as described in Materials and Methods. Panel **c** shows the mean number of CFU ± standard deviation from three independent experiments.

To rule out the possibility that AgNP-mediated inhibition of *M*.*tb*-induced immune responses resulted from a reduced infection efficiency (internalization) of *M*.*tb* in MDM in the presence of AgNP, we compared the number of MDM that internalized *M*.*tb* in the absence or presence of 10μg/mL Ag20-citrate or Ag110-citrate. No statistically significant effect of AgNP was observed in the proportions of MDM that had internalized *M*.*tb* at 2 and 4 hours (Fig 8A and 8B) compared to AgNP-unexposed MDM infected with *M*.*tb* only. In addition, no significant difference was observed in CFU numbers from lysed MDM that had been infected with *M*.*tb* in the absence or presence of Ag20-citrate or Ag110-citrate (Fig 8C). Taken together, these results indicate that the observed suppression of *M*.*tb*-induced responses in presence of AgNP (Figs 4 and 5) is not due to an AgNP-mediated reduction in the *M*.*tb* uptake by MDM.

### Interactions of AgNP with *M*.*tb*-induced TLR signaling pathways

**Fig 9:**
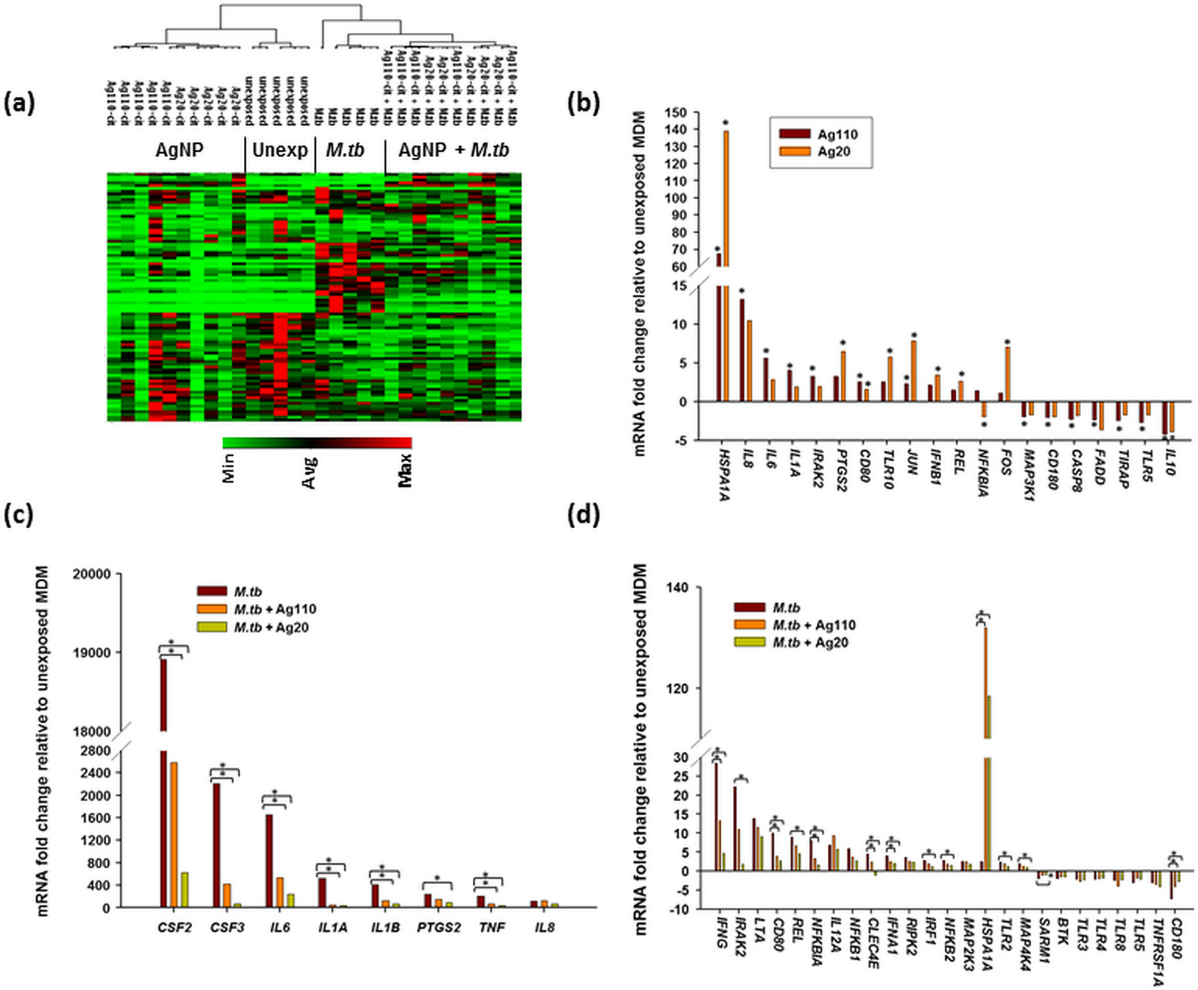
Comparison of AgNP-induced TLR signaling pathway-specific gene expression in MDM in the absence and presence of *M*.*tb*. MDM from five donors were exposed to Ag20-citrate ± *M*.*tb*, Ag110-citrate ± *M*.*tb*, *M*.*tb*, or left unexposed for 4 hours at 37°C in a humidified 5% CO_2_ environment followed by RNA extraction. The final concentration of AgNP was 10 μg/mL and *M*.*tb* was used at a MOI 10. RNA was analyzed by TLR pathway specific RT^2^ profiler arrays (Cat. No. PAHS 018E, Qiagen Sciences, MD) [28]. Levels of cDNA were calculated with the relative quantitation method (ΔΔC_t_ method) from the PCR array data using analysis software accessed from http://sabiosciences.com/pcrarraydataanalysis.php. Statistical differences in fold-mRNA expression levels between AgNP-exposed and unexposed and uninfected cells were calculated using the same software. (**a**) Non-supervised clustering of the entire dataset showing the overall pattern of expression of 84 genes across 30 MDM samples from five human subjects with each row and column representing a gene and samples, respectively. A dendrogram including all 30 samples are shown. (**b**) Mean fold-changes (≥ 2-fold) of mRNA from MDM exposed to 10 μg/mL of Ag20-citrate or Ag110-citrate relative to unexposed MDM are shown. Statistically significant changes (*p* < 0.05) relative to AgNP-unexposed MDM are marked by an asterisk (*). (**c**) and (**d**) *M*.*tb*-induced alteration of mRNA expression with mean fold-changes ≥2-fold and *p*≤0.05 relative to uninfected MDM were compared with that from MDM treated with *M*.*tb* + Ag20-citrate or *M*.*tb* + Ag110-citrate. Panel **c** shows mRNA fold-changes induced by *M*.*tb* alone that are ≥ 100-fold and panel **d** shows mRNA fold-changes induced by *M*.*tb* alone that are < 100-fold. Statistically significant changes (*p* < 0.05) relative to *M*.*tb*-induced expression are marked by an asterisk (*).

To examine whether AgNP exposure interferes with *M*.*tb*-induced activation of TLR signaling pathways in MDM, RNAs from MDM exposed to Ag20-citrate (10 μg/mL), Ag110-citrate (10 μg/mL) in the absence and presence of *M*.*tb*, and *M*.*tb* alone (0 μg/mL AgNP) were compared with those from uninfected MDM using a human TLR signaling pathway specific RT^2^ profiler array as described previously [28]. Expression profiles of 84 genes (examined by the TLR signaling pathway array) were assessed across 30 MDM samples from five study subjects clustered in four exposure groups: unexposed, AgNP-exposed, *M*.*tb*-infected and AgNP-exposed + *M*.*tb-*infected (Fig 9A). The exposure of MDM to Ag20-citrate and Ag110-citrate induced the expression of several genes related to TLR-mediated signal transduction pathways including *IL8*, *IL6*, *IL1A* and *IFNB1* mRNA, which are NF-κB target genes (Fig 9B). Interestingly, *HSPA1A* mRNA encoding the stress-inducible Hsp72 was greatly increased (*p*<0.05) in MDM exposed to both Ag20-citrate (140-fold) and Ag110-citrate (70-fold) relative to unexposed MDM (0-line) (Fig 9B).

**Fig 10:**
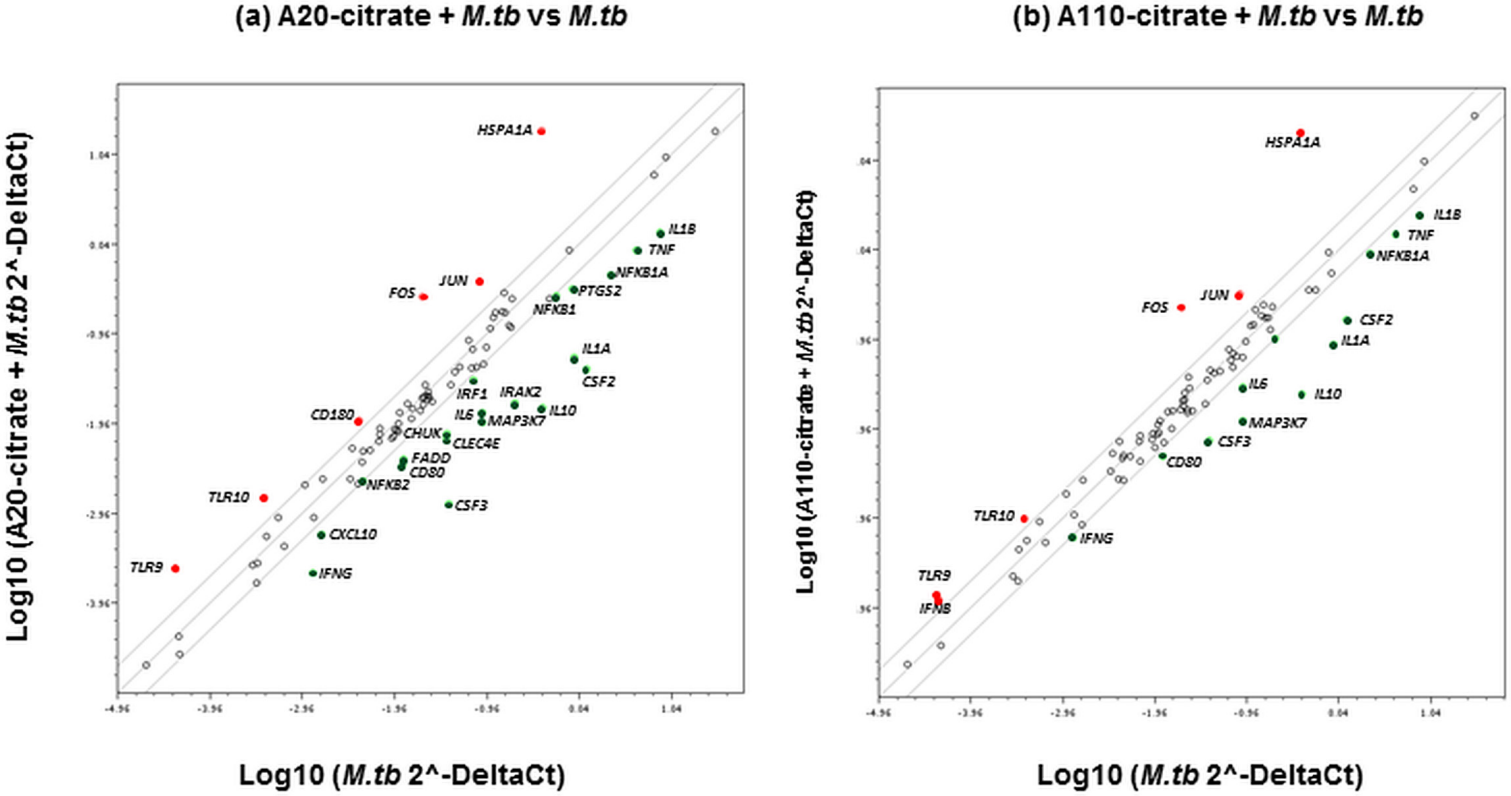
Comparison of Ag20 and Ag110 exposure effects on *M*.*tb*-induced gene expression. Scatter plots comparing the normalized expression of genes from *M*.*tb* infected MDM against that from MDM exposed to Ag20-citrate + *M*.*tb* (**a**) and Ag110-citrate + *M*.*tb* (**b**) are shown. The central lines represent unchanged gene expression. Upregulated and downregulated genes are indicated in red and green colors, respectively.

Infection of MDM with *M*.*tb* altered the expression of 33 mRNAs (out of 84 genes examined by the TLR signaling pathway array) as defined by ≥ 2-fold increases or decreases (*p* < 0.05) relative to uninfected MDM (Fig 9C and 9D). These 33 mRNAs included TLRs and TLR-interacting effectors (*IRAK2*, *TLR3*, *TLR4*, *TLR8*, and *TLR5*) and downstream targets of the TLR signaling pathway (*CSF2*, *CSF3*, *IL6*, *IL1A*, *IL1B*, *PTGS2*, *TNFA*, *IL8*, *IFNG*, *IL12A*, *NFKBA1*, *NFKB1*, *NFKB2*, *IFNA*, *LTA*, *REL*, *IRF1*, *MAPK4K4*, *MAP2K3*, *TNFRSFA1*), as well as key mediators of the TLR signaling pathway including adaptors and proteins that interact with TLRs (*HSPA1A*, *SARM1*, *BTK1*). *M*.*tb* also altered the expression of *CD80*, *RIPK2* and *CD180*, which are involved in the regulation of adaptive immunity (Fig 9C and 9D). Exposure of *M*.*tb*-infected MDM to Ag20-citrate or Ag110-citrate inhibited (*p*<0.05) the expression of several of the *M*.*tb*-induced mRNAs including *CSF2*, *CSF3*, *IL6*, *IL1A*, *IL1B*, *TNFA* and *IFNG* (Fig 9B and 9C). Effects of Ag20-citrate and Ag110-citrate on *M*.*tb*-induced mRNA expression are compared and mean fold-changes (≥ 2-fold) and related *p*-values are summarized in Table 2. Genes induced by the activation of the NF-κB pathway by *M*.*tb* are shown in Table 2 (marked by asterisks). Clearly, MDM exposure to Ag20-citrate reduced *M*.*tb*-induced gene expression to a greater extent (*p*<0.05) than MDM exposure to Ag110-citrate (Fig 10) further indicating that smaller-sized AgNP conferred greater immunomodulatory effects than larger ones.

**Table 2:** Modulation of *M*.*tb*-induced TLR pathway activation by AgNP.

Gene symbol	Ag20 + *M*.*tb*		Ag110 + *M*.*tb*	
	Fold-change	*p*-value	Fold-change	*p*-value
*FOS*	13	0.008958	11	0.056398
*HSPA1A*	47	0.000001	52	0.00033
*JUN*	4	0.000199	4	0.010921
*CD180*	3	0.000329	-	-
*TLR10*	4	0.002096	3	0.061568
*TLR9*	6	0.010852	3	0.124455
*IFNB1*	-	-	3	0.229726
*CD80*	-4	0.001071	-3	0.00722
*CHUK*	-5	0.365355	-	-
*CLEC4E*	-5	0.001016	-	-
CSF2*	-30	0.013778	-7	0.021065
CSF3*	-31	0.016669	-5	0.041353
*CXCL10*	-3	0.072188	-	-
*FADD*	-3	0.002378	-	-
IFNG*	-6	0.003102	-2	0.0245
IL10*	-27	0.000721	-17	0.000856
IL1A*	-16	0.013459	-10	0.015569
IL1B*	-6	0.004802	-3	0.01435
IL6*	-7	0.013154	-3	0.01926
*IRAK2*	-12	0.020478	-2	0.080313
*IRF1*	-2	0.014488	-	-
*MAP3K7*	-9	0.3465	-7	0.346541
NFKB1*	-2	0.074226	-	-
NFKBIA*	-5	0.000742	-2	0.003095
*PTGS2*	-3	0.01948	-	-
*TIRAP*	-2	0.009272	-	-
TNF*	-5	0.023304	-3	0.041209

The abundance of TLR pathway-specific mRNAs was compared in MDM exposed to Ag20-citrate + *M*.*tb* or Ag110-citrate + *M*.*tb* with that from MDM infected with *M*.*tb* only. Mean fold-changes of mRNA ≥ 2-fold and *p*-values relative to MDM infected with *M*.*tb* only are shown. The NF-κB target genes are marked by an asterisk (*). The *p*-values are calculated based on a Student’s t-test of the replicate 2^(- Delta Ct) values for each gene in the *M*.*tb*-infected and each of the *M*.*tb* + AgNP-exposed groups.

### AgNP-induced Hsp72 expression—inverse correlation with IL-1β expression in MDM

**Fig 11:**
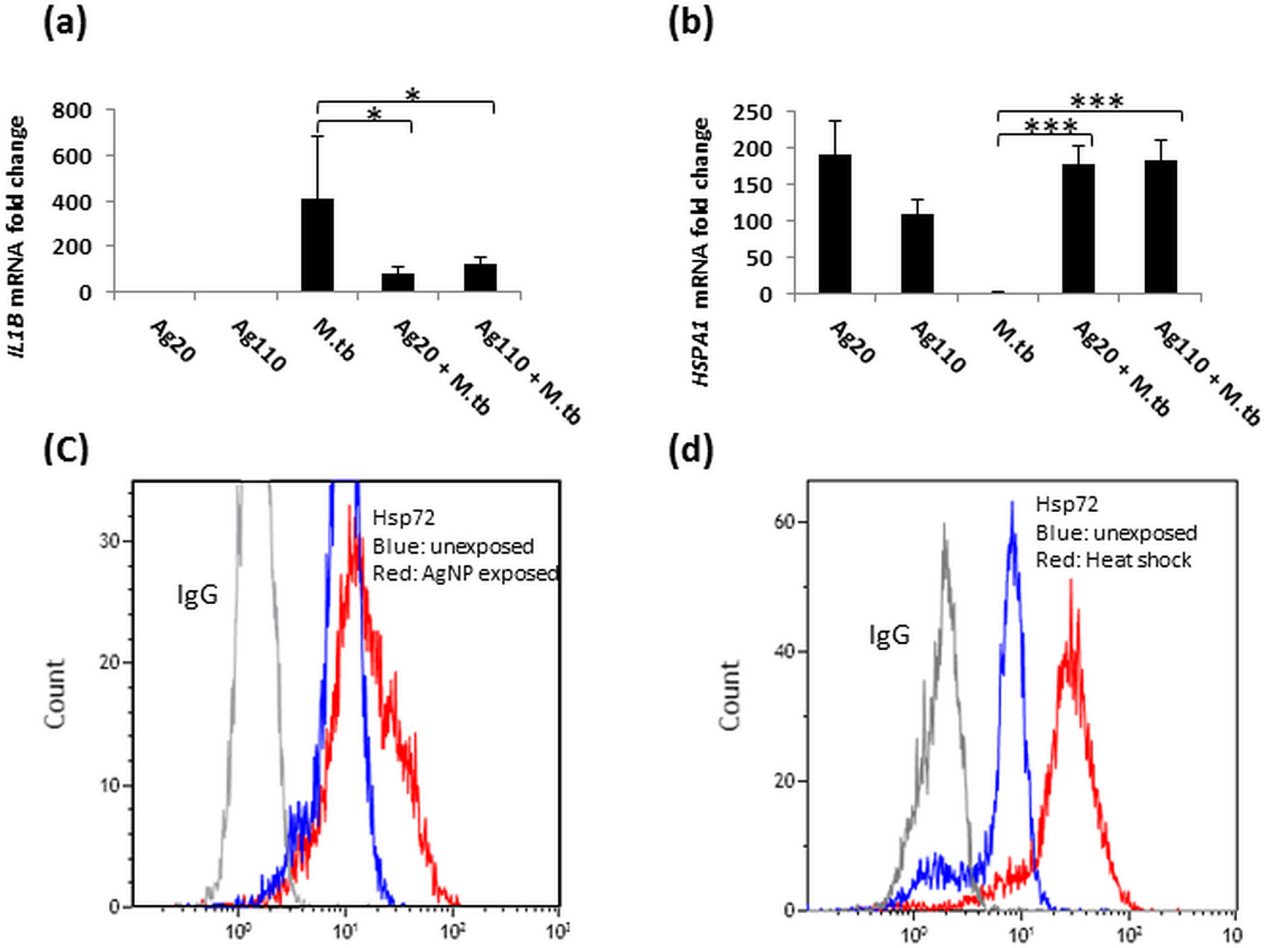
Validation of Hsp72 expression induced by AgNP in MDM. RNA from the samples used for TLR arrays (Fig 7) was used to validate array results by qRT-PCR using primers corresponding to *IL1B* (a) and *HSPA1* (b) (see Materials and Methods). Each data point (Y-axis) represents mean fold-changes ± SD from MDM of 5 independent subjects. Statistically significant changes relative to unexposed MDM are marked by * (*p* < 0.05) or *** (*p* <0.001). Unexposed (blue line) and Ag20-citrate exposed (10 μg/mL for 4 hours at 37°C at 5% CO_2_ in a humidified environment, red line) MDM were stained with anti-Hsp72 Ab or control IgG (grey line) (**c**). Data shown in (**c**) are representative of the FACS profile from one experiment, which was repeated twice using MDM from a total of three different donors. MDM exposed to 42°C heat shock for 1h followed by incubation at 37°C for 3h stained with anti-Hsp72 Ab served as a positive control (d). Blue and red lines represent the unexposed and heat shocked anti-Hsp72-stained MDM. Unexposed MDM stained with Isotype-matched IgG Ab are shown by gray lines in (**c**) and (**d)**, representative of two independent experiments).

Results of the PCR array assessments (Fig 9) were validated by qRT-PCR and flow cytometry (Fig 11). *IL1B* mRNA expression was shown to be suppressed by AgNP (Fig 9A) while AgNP induced the expression of *HSPA1* mRNA which encodes for Hsp72 (Fig 11B). Interestingly, the expression of *IL1B* mRNA was inversely correlated with that of *HSPA1* (compare Fig 11A and 11B). Increases in *HSPA1* mRNA expression corresponded to increases in Hsp72 protein production in MDM exposed to AgNP (Fig 11C). MDM exposed to heat shock are shown as a positive control (Fig 11D).

## Discussion

Innate host resistance plays a crucial role in controlling the initial *M*.*tb* infection and shaping its clinical outcome. Here we demonstrate, for the first time that the exposure of MDM to AgNP inhibits *M*.*tb*-induced expression of *IL1B*, *TNFA*, and *IL10* mRNA, cytokines of critical importance in antimicrobial immunity. Such AgNP-mediated alterations of *M*.*tb*-induced host immune responses, may potentially attenuate protective immunity against *M*.*tb* and increase risks to public health. Inhibition of cytokine mRNA expression was confirmed in assessments of *M*.*tb*-induced IL-1β protein expression, which, like the expression of IL-1β mRNA, was suppressed by AgNP. Combined, these observations are reminiscent of earlier studies from our lab showing DEP-mediated suppression of immune responses to *M*.*tb* in human PBMC [28]. Suppression of *IL1B*, *TNFA*, and *IL10* expression appeared to be specific to AgNP exposure since carbon black, an inert nanoparticle of similar size, did not inhibit *M*.*tb*-induced IL-1β protein expression (compare Fig 5A and 5B).

In early stages of *M*.*tb* infection, interactions between macrophages and *M*.*tb* leading to induction of proinflammatory cytokines are crucial, not only as components of protective innate host immunity, but also in inducing subsequent adaptive immunity [48]. Proinflammatory IL-1β and TNF-α production are instrumental in containing *M*.*tb* infection in granulomas, and in preventing dissemination of *M*.*tb*. *M*.*tb*-induced IL-1β, which functions in an autocrine fashion in human macrophages, is also pivotal in limiting intracellular *M*.*tb* growth by increasing TNF signaling and through subsequent upregulation of caspase-3 [30,44,49]. Furthermore, acute susceptibility to *M*.*tb* observed in mice deficient in either IL-1β or IL-1R indicates the importance of IL-1β signaling in protective immunity to *M*.*tb* [50]. Therefore, the observed AgNP-mediated suppression of the production of *M*.*tb*-induced proinflammatory cytokines including IL-1β (Figs 4 and 5) may lead to deregulation of efficient host immune responses to *M*.*tb*. Our data indicate that the suppression of *M*.*tb*-induced cytokine expression is neither due to microbicidal effects of AgNP (Fig 7) nor to a reduced uptake of *M*.*tb* in MDM in presence of AgNP (Fig 8).

Innate antimycobacterial host immunity is conferred to a large extent by activation of TLR2, TLR4 and TLR9 [51,52] through binding of *M*.*tb*-derived ligands such as lipoarabinomannan (LAM) and mycobacterial 19 kDa protein [53], *M*.*tb* heat shock proteins 65 and 71 [54], and mycobacterial DNA [55], respectively. TLR engagement on monocytes, alveolar macrophages, and dendritic cells [56] leads to the activation of mitogen-activated protein (MAP) kinases, transcription factors NF-κB and the interferon regulatory factor (IRF) family [57] and subsequent release of proinflammatory cytokines and chemokines [21,56,58]. Here we show that AgNP attenuate the NF-κB pathway as indicated by the down regulation of NF-κB target genes *CSF2*, *CSF3*, *IFNG*, *IL1A*, *IL1B*, *IL6*, *IL10*, *TNFA*, and *NFKB1A*, and subsequently inhibit *M*.*tb*-induced proinflammatory responses (Fig 9C and 9D). Thus, the suppression of *M*.*tb*-induced proinflammatory cytokine production may in part be a consequence of AgNP-mediated inhibition of *M*.*tb*-induced activation of TLR pathways.

We also observed a robust upregulation of *HSPA1A* mRNA encoding Hsp72 in MDM exposed to both Ag20-citrate and Ag110-citrate (Figs 9B and 11). This observation is consistent with reports showing induction of Hsp72 by AgNP both in *Drosophila melanogaster* [59] and the human alveolar type II epithelial cell line A549 [42]. Interestingly, the reported induction of Hsp72 in A549 cells appears to be specific to AgNP as exposure to TiO_2_ NP [60] did not induce Hsp72 expression. AgNP specificity of this effect is further supported by our findings that neither carbon black particles nor DEP induce the expression of Hsp72 in MDM (data not shown) or PBMC [28], respectively.

A correlation between ROS induction and expression of Hsp72 by AgNP in *Drosophila* has been reported [59]. The immunomodulatory effects of Hsp72 vary depending on its cellular localization. Extracellular Hsp72 has been reported to be immunostimulatory and proinflammatory in mammalian cells [61] including human PBMC, monocytes and macrophages [62,63] while intracellular Hsp72 is reported to be immunosuppressive [64]. However, the immunostimulatory effects of extracellular Hsp72 have been attributed to contaminating LPS in the Hsp72 preparation in a recent study [65]. Intracellular Hsp72 exerts anti-inflammatory effects by inhibiting the MAPK and NF-κB pathway [66] and blocking NF-κB-binding to the target genes leading to their activation and consequently the production of proinflammatory cytokines.

We noted an inverse correlation between *IL1B* and *HSPA1* mRNA expression in AgNP-exposed MDM that were infected with *M*.*tb* (Fig 11), which suggests that upregulation of Hsp72 mRNA (Fig 11B) and protein expression (Fig 11C) may interfere with or suppress *IL1B* expression.

**Fig 12:**
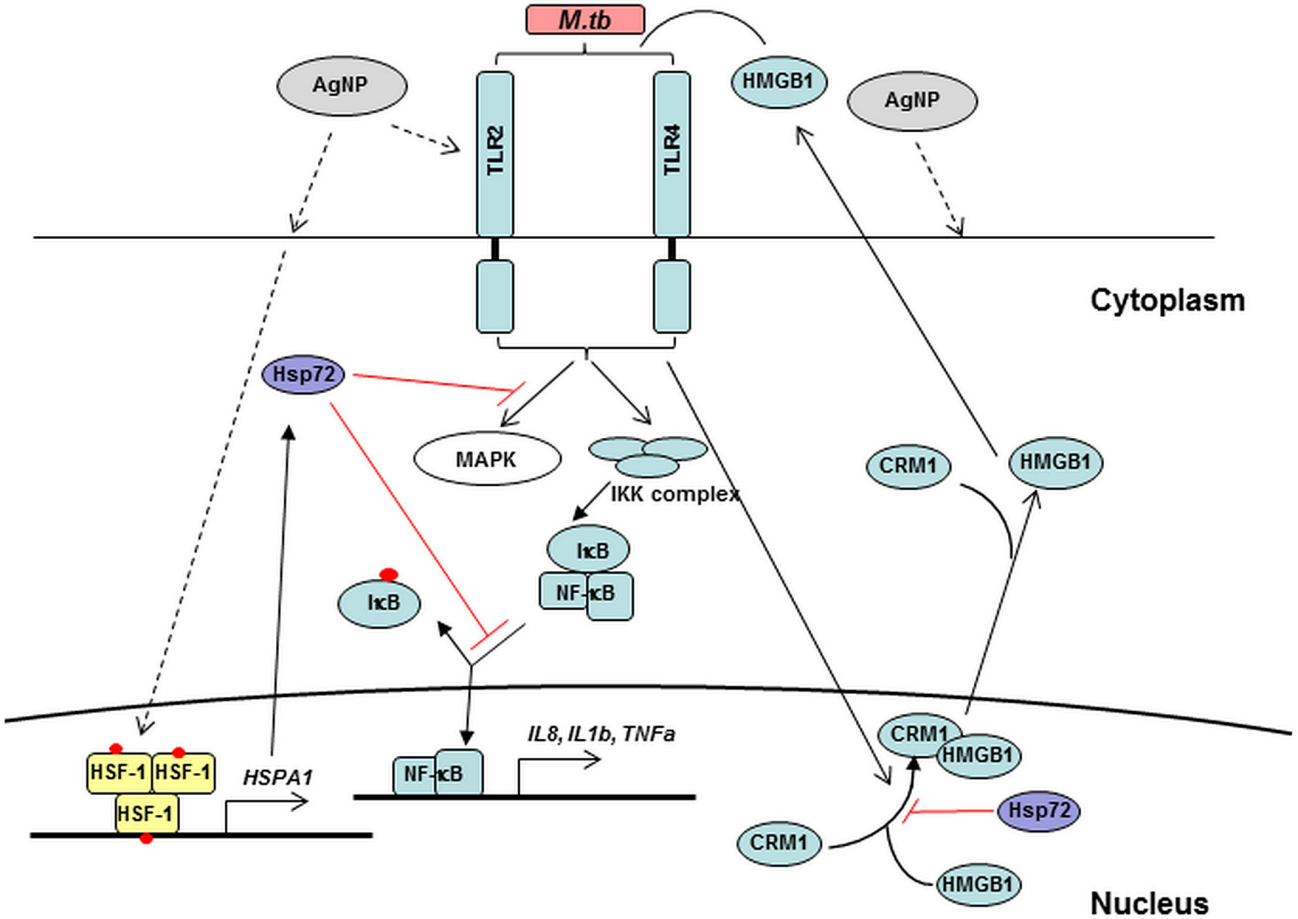
Schematic of hypothetical mechanisms of AgNP-induced suppression of *M*.*tb*-induced TLR signaling and proinflammatory responses. *M*.*tb* binds to TLRs and activates MAPK and NF-κB leading to the expression of cytokines such as IL-1β and TNF-α. IKK complex phosphorylates IκB which leads to its dissociation from the NF-κB complex followed by translocation of NF-κB into the nucleus where NF-κB binds and activates target genes. *M*.*tb* induces the cytoplasmic translocation of HMBG1 protein by chromosome region maintenance 1 (CRM1)-dependent transport and secretion. HMBG1 binds to TLR receptors and activates MAPK and NF-κB pathways [45]. AgNP, which are thought to enter cells *via* receptor-mediated endocytosis (dashed lines) upregulate the expression of *HSPA1A* that encodes Hsp72 *via* the activation and binding of heat shock factor (HSF), the major transcription factor that binds to the promoter of *HSPA1A*. Intracellular Hsp72 potentially suppresses the cytoplasmic transport and secretion of HMGB1 proteins as well as the activation of MAPK and NF-κB pathways [66] leading to the suppression of *M*.*tb*-induced cytokine expression in presence of AgNP. Hsp72 can be secreted and bind to TLR4 and activate MAPK and NF-κB pathways [88]. Red dots represent phosphorylation of HSF-1 and IκB proteins. The potential inhibitory effects of Hsp72 are shown with red solid lines. Signaling pathways that are reported to be induced by specific ligand receptor interactions and the more hypothetical pathways are shown with solid and dashed lines, respectively.

A plausible mechanism by which upregulation of Hsp72 may potentially block the expression of proinflammatory cytokines in response to external stimuli (*M*.*tb*) could be the inhibition of the cytoplasmic translocation of High-Mobility-Group-Box 1 (HMGB1), an ubiquitous non-histone nuclear protein. Overexpression of intracellular Hsp72 has been shown to strongly inhibit LPS and TNF-α-induced cytoplasmic translocation and subsequent release of HMGB1 that has been implicated in many inflammatory diseases [66,67]. Secretion of HMGB1 has been observed in *M*.*tb*-infected murine bone marrow-derived macrophages (BMDM) and bronchoalveolar cells of *M*.*tb*-infected guinea pigs. Moreover, incubation with anti-HMGB1 antibodies decreases *M*.*tb*-induced IL-1β and TNF-α production in BMDM [45] indicating the importance of HMGB1 in promoting *M*.*tb*-induced inflammatory responses. HMGB1 binds to TLR2, TLR4, TLR9 and receptor for advanced glycation end-products (RAGE) leading to the activation of downstream signaling cascades involving p38 mitogen-activated protein kinase (MAPK), c-Jun NH(2)-terminal kinase (JNK) and NF-κB implicated in the activation of NF-κB pathway in rodent and human cells [68–72]. Recently, HMGB1 was used as an adjuvant for *M*.*tb* protein ESAT6 in the design of an anti-tuberculous vaccine candidate, which induced potent cell-mediated immunity against subsequent *M*.*tb* challenges [73]. Taken together, we speculate (Fig 12) that AgNP suppress *M*.*tb*-induced host immune responses by blocking the NF-κB pathway via upregulation of Hsp72 and subsequent suppression of HMGB1 production in *M*.*tb*-infected macrophages. In addition to transcriptional activation through TLR-mediated pathways, other pathways such as *M*.*tb*-induced caspase-1 dependent inflammasome activation [47,74] as well as both TLR and caspase-1 independent mechanisms [44] are shown to be important for IL-1β production. Our schematic model involving Hsp72 (Fig 12) is speculative and requires future confirmation. Nevertheless, it resolves the apparent contradiction that AgNP alone induce the expression of IL-1β (at least at the mRNA level at 25 μg/mL dose, Fig 4) while inhibiting the *M*.*tb*-induced expression of both IL-1β mRNA and protein. Exposure of MDM to AgNP induces expression of *IL1B* as well as Hsp72, which is shown to inhibit NF-κB activation. Thus, during simultaneous exposure to AgNP and infection with *M*.*tb* of MDM, AgNP-induced Hsp72 may potentially inhibit *M*.*tb*-mediated NF-κB activation. More research is needed to provide further insight into the mechanisms of AgNP-mediated suppression of antibacterial innate immunity.

Size, charge, and surface modifications of metallic NP have been shown to affect their cytotoxicity [14,75–77] and toxicity of AgNP has been reported in *in vitro* studies [41,78,79]. However, the relative contribution of Ag^+^ ions released from AgNP or the particulate nature of the AgNP to cytotoxicity is not clear. Our data show that all AgNP studied here (Ag20-citrate, Ag20-PVP, Ag110-citrate, and Ag110-PVP) reduce the viability of primary human MDM (80% reduction over a 24-hour exposure period, Fig 3) compared to unexposed MDM or MDM exposed to PVP only, even at the lowest concentration (5 μg/mL) examined. It is noteworthy that the AgNP-mediated suppression of *M*.*tb*-induced immune responses observed in our study occurred during 4-hour exposure periods during which AgNP toxicity was not detected. In contrast to reports indicating that smaller AgNP (15–30 nm) are more toxic than larger AgNP (55 nm) [4,41,80] in murine and human cell lines, we observed no significant differences in cytotoxicity between Ag20 and Ag110 at any of the concentrations examined. Studying the same AgNP used in our study (from NCNHIR), Wang *et al*. reported that Ag20-citrate and Ag20-PVP were more cytotoxic than Ag110-citrate and Ag110-PVP in BEAS-2B and RAW cell lines [14]. Wang *et al*. also demonstrated greater Ag^+^ ion release from Ag20 (>5%) than from Ag110 in water corresponding to higher toxicity of the smaller particles. In contrast, we observed a maximal 2% Ag^+^ ion release from Ag20-citrate and negligible levels from Ag20-PVP, Ag110-citrate and Ag110-PVP when incubated for 24 hours at pH7 (Fig 2). It is of note that in order to avoid an overestimation of the amount of Ag^+^ ions released into solution, we centrifuged and filtered AgNP through a 2kDa membrane, to ensure that the Ag^+^ ions were separated from the AgNP (Materials and Methods).

Discrepancies between earlier published work and our current study findings, regardingAgNP size and coating effects on AgNP-mediated cytotoxicity, may be due to differential susceptibility of cultured cell lines vs. primary cells, such as the MDM used here. In MDM even the lowest concentration of AgNP (5 μg/mL) may have masked the differences in toxicity between AgNP with different size and surface coating. Interestingly, in contrast to AgNP, cytotoxicity of TiO_2_ NP in THP-1 cells is proportional to its particle size with the smallest size being the least toxic [41]. Thus, size, stability and chemical composition of NP have to be taken into account in the evaluation of NP cytotoxicity [81,82].

ROS production due to oxidative stress, results in cellular toxicity upon NP exposure [83] and has been proposed to be a direct or indirect cause of AgNP-induced cytotoxicity [41,79]. Nonetheless, in a study involving five different types of NP in hematopoietic and cancer cell lines, Diaz *et al*. did not observe any direct correlation between cytotoxicity and ROS production and concluded that the cytotoxicity of NP depends on the cell types in which it is studied [84]. Indeed, in addition to AgNP-induced ROS production, other mechanisms such as p38 activation, DNA damage, cell cycle arrest and apoptosis have been shown to be underlying causes of AgNP-mediated cellular toxicity [85,86].

It has been shown recently that 20 nm Ag shell on gold core particles, such as those used in this study, have a higher solubility than 20 nm pure Ag particles. Since biological properties depend on NP and the Ag^+^ ions dissolved from them, pure Ag and Ag shell on gold core particles may differ in their biological properties [87].

In summary, the current study demonstrates that AgNP exposure can potentially impair *M*.*tb*-induced activation of TLR signaling in MDM as suggested by the suppression of target gene expression downstream of TLR pathway. Hsp72-mediated inhibition of the activation of NF-κB pathway may have contributed to the observed suppression of the *M*.*tb*-induced host response in presence of AgNP. Our findings clearly establish that AgNP exposure confers a suppressive effect on *M*.*tb*-induced immune responses that in large part is due to the physicochemical properties of the AgNP not Ag^+^ ions released from the NP.

This work was performed with avirulent *M*.*tb* laboratory strain H37Ra as it was not feasible with virulent *M*.*tb* (which causes clinical TB) under BSL-3 safety work conditions at present. Nonetheless, the current study addresses the less explored question whether AgNP exposure affects host immune defenses against infectious pathogens with complex pathogenesis such as that of *M*.*tb*.

The emergence of AgNP as one of the most commonly used NP in consumer products may increase the risk of human exposures that have the capacity to significantly alter inflammatory immune responses required for the abrogation of bacterial infections.

## Supporting Information

S1 Fig Phenotypic characterization of MDM. Expression of markers CD11c (a), HLA-DR (b), CD11b (c), CD14 (d), CD16 (e), and CD163 (f) on the surface of MDM was evaluated by flow cytometry with a Gallios Flow Cytometer (Beckman Coulter, Miami, FL) and analyzed with Kaluza Analysis Software (Beckman Coulter) for expression of macrophage-specific surface markers. All cell surface markers and isotype-matched monoclonal antibodies are indicated by green and black lines, respectively.
(TIF)

S1 Table Phenotypic characterization of human MDM. Expression of macrophage surface markers on MDM used in this study was characterized by flow cytometry. Percent positive MDM obtained from adherent monocytes after 7-days of differentiation were evaluated for macrophage/monocyte, T and B lymphocyte markers by surface staining with monoclonal and isotype-matched control antibodies. Proportions of CD19 and CD3 positive cells contaminating the MDM population were < 0.1% (data not shown).
(DOCX)

S1 Data Raw data for Fig 1. (7Z)

S2 Data Raw data for Fig 2. (XLSX)

S3 Data Raw data for Fig 3. (XLS)

S4 Data Raw data for Fig 4. (XLS)

S5 Data Raw Data for Fig 5. (XLS)

S6 Data Raw Data for Fig 6. (XLSX)

S7 Data Raw Data for Fig 7. (XLS)

S8 Data Raw Data for Fig 8. (XLSX)

S9 Data Raw Data for Fig 9. (XLS)

S10 Data Raw Data for Fig 11. (XLS)
