# Supplementary figures and images for: Modulation of Human Macrophage Responses to Mycobacterium tuberculosis by Silver Nanoparticles of Different Size and Surface Modification
Source: PLoS One. 2015 Nov 18;10(11):e0143077. doi: 10.1371/journal.pone.0143077 (PMC4651328; doi:10.1371/journal.pone.0143077)

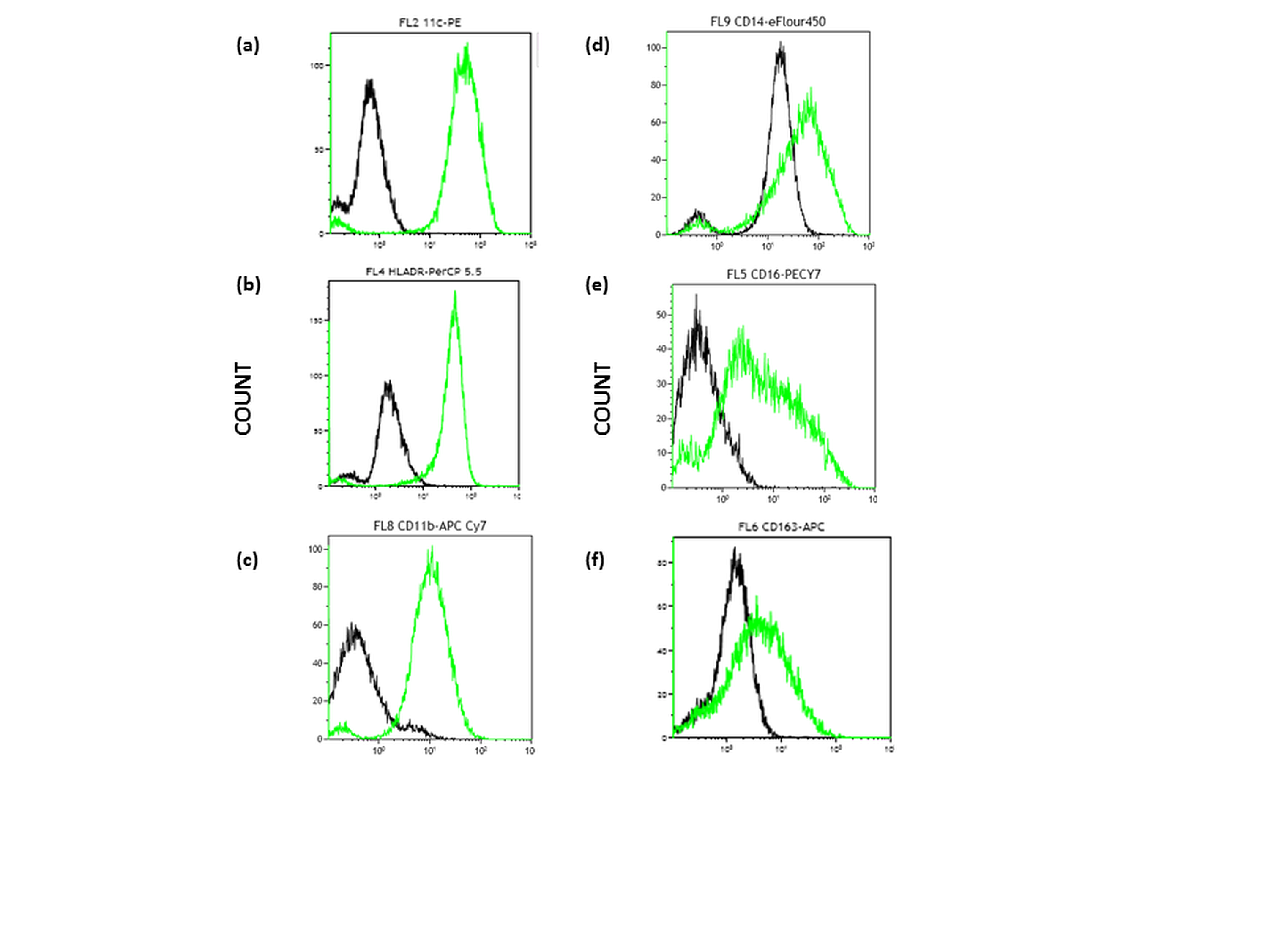

Supplement: S1 Fig — Expression of markers CD11c (a), HLA-DR (b), CD11b (c), CD14 (d), CD16 (e), and CD163 (f) on the surface of MDM was evaluated by flow cytometry with a Gallios Flow Cytometer (Beckman Coulter, Miami, FL) and analyzed with Kaluza Analysis Software (Beckman Coulter) for expression of macrophage-specific surface markers. All cell surface markers and isotype-matched monoclonal antibodies are indicated by green and black lines, respectively. (TIF) [file pone.0143077.s001.tif]
