# Supplementary material for: Modulation of Human Macrophage Responses to Mycobacterium tuberculosis by Silver Nanoparticles of Different Size and Surface Modification
Source: PLoS One. 2015 Nov 18;10(11):e0143077. doi: 10.1371/journal.pone.0143077 (PMC4651328; doi:10.1371/journal.pone.0143077)
Supplement: S1 Table — Expression of macrophage surface markers on MDM used in this study was characterized by flow cytometry. Percent positive MDM obtained from adherent monocytes after 7-days of differentiation were evaluated for macrophage/monocyte, T and B lymphocyte markers by surface staining with monoclonal and isotype-matched control antibodies. Proportions of CD19 and CD3 positive cells contaminating the MDM population were < 0.1% (data not shown). (DOCX) [file pone.0143077.s002.docx]

**S1 Table. Phenotypic Characterization of Human MDM**

| **Antibody** | **Antibody positive Cells (%)** | **Isotype antibody positive cells (%)** | **Unstained cells (%)** |
| --- | --- | --- | --- |
| CD11c | 96.5 | 0.4 | 0.2 |
| HLA-DR | 93 | 0.7 | 0.1 |
| CD16 | 59.4 | 2 | 0 |
| CD163 | 32.8 | 0.6 | 0.6 |
| CD11b | 86.7 | 3.6 | 0 |
| CD14 | 35.9 | 0.6 | 0.6 |
